# Guiding waves through chaos: Universal bounds for targeted mode transport

**DOI:** 10.1126/sciadv.aeb1158

**Published:** 2026-01-28

**Authors:** Cheng-Zhen Wang, John Guillamon, Ulrich Kuhl, Matthieu Davy, Mattis Reisner, Arthur Goetschy, Tsampikos Kottos

**Affiliations:** ^1^ Wave Transport in Complex Systems Lab, Department of Physics, Wesleyan University, Middletown, CT 06459, USA.; ^2^ Université Côte d’Azur, CNRS, Institut de Physique de Nice (INPHYNI), 06200 Nice, France.; ^3^ Université de Rennes, CNRS, IETR, UMR 6164, F-35000 Rennes, France.; ^4^ ESPCI Paris, PSL University, CNRS, Institut Langevin, Paris, France.

## Abstract

Controlling wave propagation in complex environments is a central challenge across wireless communications, imaging, and acoustics, where multiple scattering and interference obscure direct transmission paths. Coherent wavefront shaping enables precise energy delivery but typically requires full knowledge of the medium. Here, we introduce a universal statistical framework for targeted mode transport (TMT) that circumvents this limitation and validate it on various platforms including microwave networks, two-dimensional chaotic cavities, and three-dimensional reverberation chambers. TMT quantifies the efficiency of transferring energy between specified input and output channels in multimode wave-chaotic systems. We develop a diagrammatic theory that predicts the eigenvalue distribution of the TMT operator and identifies the macroscopic parameters—coupling strength, absorption, and channel control—that govern performance. The theory provides explicit bounds for optimal TMT wavefronts and captures phenomena like statistical transmission gaps and reflectionless states. These findings establish design principles for energy delivery and information transfer in complex environments, with broad implications for adaptive signal processing and wave-based technologies.

## Teaser

A universal framework reveals governing laws for optimal wave focusing and maximal energy delivery in chaotic environments.

## INTRODUCTION

Delivering energy or information through complex enclosures, such as reverberant electromagnetic environments, is complicated due to multiple scattering, modal overlap, and sensitivity to small perturbations. These features, although fundamental to chaotic wave dynamics, severely limit the effectiveness of conventional communication or energy transfer strategies (*1*, *2*). Over the past decade, coherent wavefront shaping (CWS) has emerged as a powerful approach to exploit rather than avoid this complexity (*3*–*5*). By tailoring the spatial or spectral structure of the input field, CWS can focus or steer waves deep into scattering systems. However, most implementations rely on detailed knowledge of the medium’s scattering matrix and assume a stable environment (*6*–*13*). In realistic environments, such as indoor wireless settings or enclosed industrial spaces, even small temporal or spatial variations in system geometry, frequency, or antenna coupling can lead to large changes in wave behavior, undermining deterministic control.

Moreover, existing CWS methods typically optimize local field enhancements or global transmission (*3*–*5*) but do not directly address the task of targeted mode transport (TMT): the coherent delivery of energy from a specific subset of input channels to a disjoint set of output channels. This task is central to applications in wireless Multiple-Input Multiple-Output (MIMO) systems, imaging, and energy routing but remains largely unexplored under realistic constraints like losses (*14*–*17*), imperfect antenna coupling (*18*–*20*), and limited access to input/output modes (*21*, *22*).

To clarify the challenge, consider a simple yet fundamental question: In a reverberant room with 10 antennas, if one can control the phases and amplitudes on 4 of them, what is the maximum energy that can be delivered to 3 others? Would the result be notably different if we targeted 6 others instead? Such scenarios are increasingly relevant in practice, yet no general framework exists to answer them without detailed modeling.

In this work, we develop a statistical theory for the TMT operator that provides such a framework. The TMT operator quantifies the efficiency of energy transfer from one subspace of input channels to a distinct set of output channels in multimode wave-chaotic systems. Although similar transport operators have been studied in diffusive media (*22*–*27*), our focus is on chaotic cavities, where the system size is comparable to the mean free path and ergodicity arises primarily from the hypersensitive nature of classical ray scattering from complex boundaries, not from disorder-induced diffusion (which is typically characterized by the ratio between the mean free path and the system size).

Using a diagrammatic approach, we derive the statistical distribution of the TMT eigenvalues and identify the key macroscopic parameters that control TMT efficiency. These include the degree of channel control, the antenna-medium coupling, and internal losses. Our predictions are independent of the microscopic details of the system, enabling universal, system-agnostic design principles. Our framework further reveals nonintuitive strategies for improving TMT, such as tuning the number of controlled channels or adjusting coupling asymmetries to maximize delivery performance.

We validate this theory through full-wave simulations and experiments in quasi-one-dimensional (1D) microwave networks, 2D chaotic cavities, and 3D reverberation chambers. In all cases, the observed transport behavior is captured by our statistical formalism, demonstrating that optimal targeted energy delivery is governed by macroscopic system properties.

By harnessing rather than mitigating the richness of multimode interference, the TMT framework opens exciting possibilities for robust and scalable wave control in complex media. Its generality makes it broadly applicable to fields including wireless communication, directed microwave energy delivery, ultrasonic imaging in heterogeneous tissues, and seismic wave control in geophysical environments.

## RESULTS

### Physical platforms for TMT

**Fig. 1.**
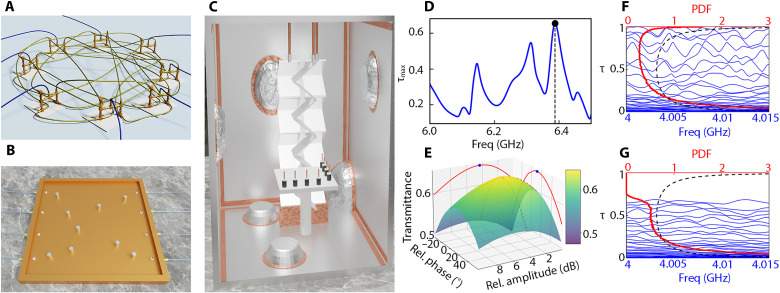
TMT across physical platforms and the effects of losses in the TMT eigenvalue statistics. (**A**) Quasi-1D complex network of coaxial cables. (**B**) 2D microwave chaotic cavity. (**C**) 3D reverberation chamber. (**D**) Experimental maximum eigenvalue of the TMT matrix as a function of input wave frequency for a network with *M* = 4 channels. The TMT process involves two input channels and one targeted output channel. The frequency at which the overall maximum TMT occurs within the analyzed frequency range is *f* ≈ 6.39 GHz, indicated by the dashed vertical line. (**E**) Corresponding transmittance of the network used in (D) as a function of the relative amplitude and phase of a two-port injected wavefront at the TMT frequency *f* ≈ 6.39 GHz. (**F**) Simulated TMT eigenvalues versus frequency (blue lines) for a network of 300 vertices coupled to *M* = 80 channels with *M*_in_ = 40 input channels and *M*_tar_ = 40 targeted channels. The coupling parameter is Γ ≈ 0.49. Each vertex is coupled to six other, randomly chosen vertices, with bond lengths randomly sampled from a uniform distribution in the range [0.1 m, 0.4 m]. The probability density function PDF (red line) is generated over frequencies in the range [3.5 GHz, 4.5 GHz]. The bimodal distribution is also shown for comparison (black dashed line). (**G**) Same plot as (F), but with uniform loss added to the cables, represented by an imaginary part of the refractive index *n*_*i*_ = 2 × 10^−3^.

The experimental platforms used for the statistical analysis of TMTs included complex networks and 2D and 3D chaotic cavities, as shown in Fig. 1 (A to C), respectively. Complex networks of coupled coaxial microwave cables (Fig. 1A) have proven to be both simple and versatile platforms for experimentally demonstrating and theoretically analyzing wave phenomena in systems with underlying classical chaotic dynamics (*28*–*33*). These networks are frequently used as models for mesoscopic quantum transport, sound propagation, and electromagnetic wave behavior in complex interconnected structures such as buildings, ships, and aircrafts (*34*–*39*). The scattering matrix **S** was measured by connecting the network to transmission lines (TLs), which were coupled to the ports of a vector network analyzer (VNA). To enable statistical processing of TMTs, multiple network configurations were generated by scanning the interrogation frequency over the range [1.5 GHz, 4.5 GHz] and systematically exploring all possible configurations among the available channels for each TMT scenario (for details, see Materials and Methods and section S1).

To further test our theory on the statistical properties of TMTs in complex systems, we conducted additional experiments using 2D chaotic cavities (see Fig. 1B; for details, see Materials and Methods). Metallic cylinders were placed at random positions inside the cavity, and the scattering matrix was measured using a VNA connected to matched coax-to-waveguide antennas attached to the cavity. A statistical ensemble has been produced by tuning the frequency in the range [8 GHz, 15 GHz] and creating all possible configurations among the available channels.

Last, we analyzed the TMT statistics in 3D chaotic enclosures (reverberation chambers; Fig. 1C). Here, the scattering matrix was measured using commercial Wi-Fi antennas. The reverberation chamber was equipped with two mechanical stirrers, one horizontal and one vertical, allowing us to generate a random ensemble of scattering matrix configurations (for details, see Materials and Methods). Additional statistics were generated by measuring the matrix **S** over the frequency range [2 GHz, 3 GHz].

In all these systems, the TMT process is characterized by the efficient coupling of a specific subset of scattering channels, controlled by another subset among the *M* available channels. The portion of the total scattering matrix describing the TMT process is given by $\tilde{S}={\text{P}}_{\mathrm{tar}}SP_{\mathrm{in}}$, where ${\text{P}}_{in}$ and ${\text{P}}_{\mathrm{tar}}$ are $M\times M_{\text{in}}$ and $M_{\text{tar}}\times M$ projection matrices. These matrices define the subspaces of $M_{\text{in}}$ controlled input channels and $M_{\text{tar}}$ targeted output channels, respectively. Here, we require that these subspaces are distinct, as is typical in wireless communication protocols, which is expressed by the orthogonality condition ${\text{P}}_{tar}\cdot P_{in}=0$.

For an incident wavefront $|\psi_{\text{in}}\rangle$ confined to the ${\text{P}}_{in}$ subspace, the outgoing signal measured in the ${\text{P}}_{tar}$ subspace after propagation through the complex multimode cavity is $\langle \psi_{\text{in}}|{\tilde{S}}^{†}\tilde{S}|\psi_{\text{in}}\rangle$. Consequently, the eigenvalues τ of the TMT matrix $T={\tilde{S}}^{†}\tilde{S}$ govern the efficiency of the process. Specifically, the extremal eigenvalues (and their corresponding eigenvectors) represent the maximum and minimum achievable TMT processes in such setups, enabling the design of wavefront schemes with extreme transport characteristics.

An example of a TMT wavefront for the system in Fig. 1A, connected to *M* = 4 antennas, is illustrated in Fig. 1 (D and E). Here, the wavefront is designed to inject an incident wave into the network via antennas α = 1, 2, aiming to maximize the transmittance to the targeted port α = 3. Because of ohmic losses in the coaxial cables and nonideal coupling between the antennas and the network, the maximum achievable transmittance for this TMT process is τ_max_ ≈ 0.65, occurring at *f* ≈ 6.39 GHz (see Fig. 1D). This is achieved by injecting a wavefront at ports α = 1, 2 with the relative amplitude and phase determined by the eigenvector components of the 2 × 2 TMT matrix **T**, as shown in Fig. 1E.

The number of controlled ($M_{\text{in}}$) and targeted ($M_{\text{tar}}$) channels, along with imperfect coupling and inherent losses, impose an upper limit on the efficiency of optimal TMT processes. This challenge is particularly pronounced in systems with many channels (*M*≫ 1), where the statistical behavior of the eigenvalues of the TMT matrix is governed by complex correlations, as illustrated by the blue lines in Fig. 1 (F and G). To gain deeper insight into this phenomenon, we conducted extensive simulations of wave dynamics in random networks and cavities and developed an analytical framework to describe the eigenvalue distribution of the TMT matrix, as detailed below.

### Statistical theory and wave simulations

To efficiently solve the wave equation for random networks and multimode cavities, we consider systems with *N* ≫ 1 modes (or vertices) coupled to *M* TLs that are used to inject and receive monochromatic waves of frequency ω. The coupling is characterized by a set of parameters ${\text{γ}}_{\alpha }$ (α=1,…,M). The incident $|\psi_{\text{in}}\rangle$ and outgoing $|\psi_{\text{out}}\rangle$ waves are related by the equation $|\psi_{\text{out}}\rangle =\text{S}|\psi_{\text{in}}\rangle$. For both complex networks and chaotic cavities, the scattering matrix **S** can be expressed as (*40*)

$$\text{S}\left(\text{ω}\right)=-\text{I}+2i{\text{D}}^{†}\frac{1}{\text{M}\left(\text{ω}\right)+i{\text{DD}}^{†}}\text{D}$$ (1)

where **D** is the coupling matrix with elements ${\text{D}}_{n\text{α}}=\sqrt{{\text{γ}}_{\alpha }}$ for a mode *n* coupled to a TL, and ${\text{D}}_{n\text{α}}=0$ otherwise. The coupling strength with the TLs is also characterized by the parameters ${\text{Γ}}_{\alpha }=1-{|\langle {\text{S}}_{\text{αα}}\rangle |}^{2}$, with ${\text{Γ}}_{\alpha }=1$ indicating a perfect (impedance-matched) coupling. Last, M(ω) represents the internal Hamiltonian dynamics within the complex isolated (${\text{γ}}_{\alpha }=0$) system.

Complex networks and cavities differ in their internal matrix M(ω) and coupling matrix **D**. For cavities, $M(\text{ω})=\text{ω}1-H_{0}$ where the *N* × *N* effective Hamiltonian ${\text{H}}_{0}$ represents the wave propagation inside the isolated cavity. In general, ${\text{H}}_{0}$ is non-Hermitian due to (ohmic or radiative) losses within the cavity. These losses are modeled by introducing an imaginary part ${\text{γ}}^{\prime }$ (loss rate) in the diagonal elements of ${\text{H}}_{0}$. For chaotic cavities, ${\text{H}}_{0}$ is statistically modeled as a random matrix taken from the Gaussian orthogonal ensemble (GOE) with elements that are drawn from a Gaussian distribution, with the variance given by $\langle {\left({\text{H}}_{0}\right)}_{\mathrm{nm}}^{2}\rangle =\frac{1}{N}\left(1+{\text{δ}}_{\mathrm{nm}}\right)$ (in units of a central frequency $\omega_{0}$ around which the measurements are performed). Furthermore, the coupling considered in this work is of the form $\gamma_{\alpha }=\gamma$ for all TLs, so that $\Gamma =1-\frac{{\left(1-\gamma \right)}^{2}}{{\left(1+\gamma \right)}^{2}}$ (*41*).

In contrast, for complex networks, M(ω) explicitly depends on the adjacency matrix of the network (see Materials and Methods), which need not be fully connected, as well as on the losses introduced through the imaginary part of the refractive index within the cables. The matrix **D** is defined such that $\gamma_{\alpha }=1$ when TL α is attached to a node. Note that, in this case, traces of system-specific features (e.g., scar effects or Anderson localization) may appear in the distribution of TMT eigenvalues and are not captured by the universal diagrammatic approach. Nevertheless, we will show that, even under these conditions, the overall qualitative agreement with the diagrammatic predictions remains strong. The estimated optimal TMT bounds and the upper edge of the transmission gap are well captured by our theory.

In Fig. 1 (F and G), we presented numerical results for the distribution P(τ) of TMT eigenvalues (red line) for a network operating in the range [3.5 GHz, 4.5 GHz]. The network consists of *N* = 300 vertices, each randomly connected to 6 others, on average, via coaxial cables with random lengths uniformly chosen in the range [0.1 m, 0.4 m]. The total numbers of controlled and targeted channels are *M*_in_ = 40 and *M*_tar_ = 40, selected from *M* = 80 available channels. In this example, the imperfect coupling to the TLs is characterized by Γ ≈ 0.49. The distribution P(τ) is compared with the well-known bimodal prediction $P\left(\text{τ}\right)=\frac{1}{\pi }\frac{1}{\sqrt{\text{τ}\left(1-\text{τ}\right)}}$ (black dashed line), which corresponds to a symmetric TMT process ($M_{\text{in}}=M_{\text{tar}}\gg 1$) under conditions of perfect coupling (Γ = 1) and no absorption (*42*). Figure 1F demonstrates that imperfect coupling skews the distribution toward smaller τ values while preserving the maximum transmittance τ_max_ ≈ 1. Conversely, the impact of absorption, shown in Fig. 1G, compresses the spectrum of eigenvalues, pushing the entire distribution toward smaller τ values. This leads to an unimodal structure in P(τ) and a reduced maximum transmittance, τ_max_ < 1.

To gain deeper insight into the parameters governing the distribution P(τ) in the limit where $M_{\text{in}},M_{\text{tar}}\gg 1$, we turn to an analytical approach. In the simplest case of perfect coupling (Γ = 1) and no absorption, all scattering channels of the matrix ***S*** are statistically equivalent. Under these conditions, the filtered random matrix (FRM) theory (*21*), which has been successfully implemented in various disordered systems in recent years (*22*, *23*, *43*–*46*), can be applied directly to the matrix ***S*** (see section S2.B). In the regime of a small number of controlled channels (*m*_in_ ≪ 1), the distribution P(τ), parametrized by the ratios $m_{\text{in}}=M_{\text{in}}/M$ and $m_{\text{tar}}=M_{\text{tar}}/M$, is concentrated around its mean $\langle \text{τ}\rangle =m_{\text{tar}}$ with a finite support $\left[{\text{τ}}^{-},{\text{τ}}^{+}\right]$, where ${\text{τ}}^{-}>0$ and ${\text{τ}}^{+}<1$. As $m_{\text{in}}$ increases, the distribution begins to spread and eventually reaches the upper limit ${\text{τ}}^{+}=1$ when the complementary channel condition (CCC) $m_{\text{in}}+m_{\text{tar}}=1$ is satisfied. Under this condition, ${\text{τ}}^{-}>0$ for all $m_{\text{tar}}$ except for the symmetric case $m_{\text{in}}=m_{\text{tar}}=1/2$, where the bimodal distribution is recovered.

The more complex and realistic scenario of imperfect coupling and finite absorption cannot be addressed using the same FRM formalism. This is primarily because selecting a subset of injection channels disrupts the equivalence among the outgoing channels of the scattering matrix S when Γ ≠ 1. In particular, the matrices ${\text{P}}_{\text{in}}$ and ${\text{P}}_{\text{tar}}$ are no longer statistically equivalent under the orthogonality constraint ${\text{P}}_{\text{tar}}\cdot {\text{P}}_{\text{in}}=0$. To address these scenarios, which frequently arise in wireless communication frameworks, a diagrammatic approach is required (see sections S2.A and S2.C). In the limit $M_{\text{in}},M_{\text{tar}}\gg 1$, we derive the distribution as $P\left(\text{τ}\right)=-\frac{1}{\pi }{\text{lim}}_{\text{η}\to 0^{+}}\text{Im}\left[g\left(\text{τ}+i\text{η}\right)\right]$, where the resolvent *g*(*z*) of the TMT operator T is expressed as

$$g\left(z\right)=\frac{1}{z}\frac{1-\left(1-\Gamma \right)\Sigma_{\text{in}}\Sigma_{\text{tar}}}{1-\left(1-\Gamma \right)\Sigma_{\text{in}}\Sigma_{\text{tar}}-{\Gamma \Sigma }_{\text{in}}/\sqrt{z}}$$ (2)

The terms Σ_in_ and Σ_tar_ are complex self-energy elements that encapsulate the effects of multiple scattering within the complex medium. These terms account for partial reflections induced by the effective barriers at the interfaces between the inner medium and the TLs, as well as absorption effects during propagation. They are determined as solutions of two coupled nonlinear equations (see Materials and Methods), only parameterized by the channel ratios $m_{\text{in}}$ and $m_{\text{tar}}$, the coupling strength Γ and the absorption factor $a=4\left(N/M\right)\gamma^{\prime }$. The latter is related to the total absorption $A\equiv 1-\langle \text{Tr}\left({\text{S}}^{†}\text{S}\right)\rangle /M$ as A=a/(1+a/Γ) (see section S2.D). Such a physically relevant parameter is not taken into account in a recent work (*47*) where the density of transmission eigenvalues has been rigorously derived for chaotic cavities described by random matrices ${\text{H}}_{0}$ with elements given by a general distribution and within the constraint of the CCC. We note, in particular, that the distribution P(τ) is not parameterized by the mean free path, the system size, or the spatial dimension, in contrast to predictions established for open diffusive systems (*23*, *24*, *46*).

**Fig. 2.**
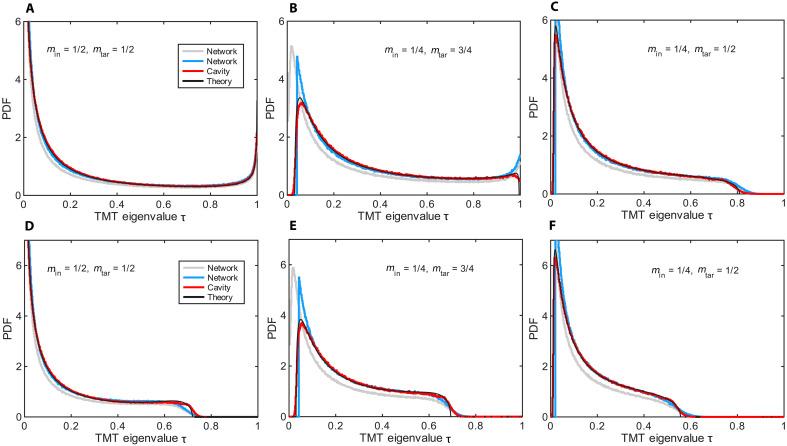
Comparison of TMT eigenvalue distributions from diagrammatic theory and simulations for complex network and cavity models, with and without losses and varying degrees of channel control. Probability density function (PDF) of TMT eigenvalues τ evaluated from wave simulations of a complex network (gray lines) and a cavity model (red lines). The blue lines indicate the PDF of the TMT eigenvalues of the network system after removing the lowest 18% of eigenvalues, which are associated with suppressed transmission due to semiclassical effects (scarring) and other localization phenomena specific to the network model (*32*, *33*). The ensemble has been generated from random configurations of cable lengths uniformly distributed in the interval [0.1 m, 0.4 m] over the frequency range [3.5 GHz, 4.5 GHz] for the networks and from random GOE matrices for the cavity model. (**A** to **C**) TMT eigenvalue distributions for lossless systems with varying input ($m_{\text{in}}$) and output ($m_{\text{tar}}$) channel ratios. (**D** to **F**) TMT eigenvalue distributions in the presence of losses for the same systems. For the complex network, the imaginary part of the cable refractive index is 2 × 10^−3^, corresponding to absorption *A* ≈ 0.13. For the lossy cavity model, the loss in the diagonal elements of the Hamiltonian matrix is γ′ = 0.016 corresponding to the same *A* ≈ 0.13. In all cases, the complex network consists of 300 vertices and is attached to 80 TLs. Each vertex is randomly coupled to six others. The cavity model consists of 300 modes and is attached to 80 TLs. Both systems are characterized by a coupling parameter Γ ≈ 0.49. In all subfigures, the predictions of the diagrammatic theory are shown (black lines). In (D) to (F), the absorption factor is *a* ≈ 0.17.

The analytical predictions for P(τ) based on Eq. 2 are shown as black lines in Fig. 2 for various $m_{\text{in}},m_{\text{tar}}$ configurations. For comparison, the same figure also includes simulation results for a complex microwave network (blue lines) and a 2D chaotic cavity (red lines), both coupled to M=80 TLs. Figure 2 (A to C) corresponds to lossless systems, whereas Fig. 2 (D and F) account for uniform losses. The microwave network simulations were performed with the same parameters as in Fig. 1 (F and G), except for $M_{\text{in}}$ and $M_{\text{tar}}$. To demonstrate the universality of our predictions, which depend only on a few macroscopic parameters and not on the microscopic details of the system, we also analyzed chaotic cavities with the same coupling parameter $\Gamma =1-{|\langle {\text{S}}_{\alpha \alpha }\rangle |}^{2}\approx 0.49$. Similarly, for the lossy case, the loss rate $\gamma^{\prime }=0.016$ in the chaotic cavity was tuned to match the total absorption, *A* ≈ 0.13, of the network. In the network simulations, ohmic losses were modeled by including an imaginary part in the refractive index, *n*_*i*_ = 2 × 10^−3^.

Figure 2 demonstrates that random networks and chaotic cavities, despite their structural differences, exhibit a distribution P(τ) for the nonzero TMT eigenvalues whose main characteristics are well captured by the diagrammatic approach. The agreement is particularly notable when small eigenvalues—associated with localization effects in sparsely connected networks (i.e., networks with low vertex valency) or system-specific phenomena such as scars, which can inhibit the development of fully ergodic dynamics (*28*–*33*)—are excluded from the analysis (see blue lines in Fig. 2). We stress that the diagrammatic approach provides, in all cases, excellent predictions for the optimal TMT statistical bound and upper transmission gap.

In greater detail, Fig. 2 (A and B) represents scenarios under the CCC ($m_{\text{in}}+m_{\mathrm{tar}}=1$), differing only in the asymmetry between the interrogating and targeted channels. Under this condition, the bimodal statistics expected for ideal coupling are skewed toward smaller transmittance eigenvalues. The scattering process still supports open channels, with τ_max_ ≈ 1, although each of the 80 TLs individually reflects ~50% of the injected signal. This constructive interference effect can be viewed as an ergodic analog of the resonance condition found in simple geometries, such as Fabry-Pérot cavities. For $m_{\text{in}}=m_{\text{tar}}=1/2$ and no absorption (Fig. 2A), Eq. 2 simplifies to an explicit solution, $P\left(\tau \right)=\frac{1}{\pi }\frac{\Gamma \left(2-\Gamma \right)}{\sqrt{\tau \left(1-\tau \right)}\left(\Gamma^{2}-4\Gamma \tau +4\tau \right)}$, whose support covers the full range τ ∈ [0, 1] (see section S2.C).

A noteworthy difference between Fig. 2 (A and B) is the marked suppression of P(τ) near τ ∼ 0 when *m*_in_ ≠ *m*_tar_, accompanied by an enhancement elsewhere, whereas τ_max_ remains very close to unity. This implies that 100% reflection is statistically improbable when the number of input channels differs from the number of remaining (target) channels. This effect is reminiscent of what is found in simpler geometries, such as Fabry-Pérot cavities or Bragg stacks with a limited number of layers, with the notable distinction that ergodicity introduces a difference between reflection gap and transmission gap only when $m_{\text{in}}\neq m_{\text{tar}}$. Furthermore, when the CCC is broken ($m_{\text{in}}+m_{\text{tar}}<1$), open channels are strongly suppressed, leading to a transmission gap near τ ∼ 1 in the P(τ) distribution and the breakdown of its bimodal structure, as shown in Fig. 2C.

Including ohmic losses further accentuates the gap in P(τ), even under the CCC, as illustrated in Fig. 2 (D and E). Despite this, the bimodal structure observed in Fig. 2 (A and B) remains. The emergence of the gap near τ ∼ 1 can be qualitatively understood by considering that losses effectively introduce additional uncontrolled output channels. However, unlike perfectly coupled uncontrolled channels, absorption channels partially backscatter the outgoing waves into the cavity (see section S2.A). Violation of the CCC primarily affects open channels (i.e., those with τ ≈ 1), whereas absorption has a more uniform impact across all channels. In the combined presence of the broken CCC and absorption, the bimodal nature of P(τ) is entirely lost, and the gap is further enlarged, as seen in Fig. 2F.

### Comparison with experimental measurements

**Fig. 3.**
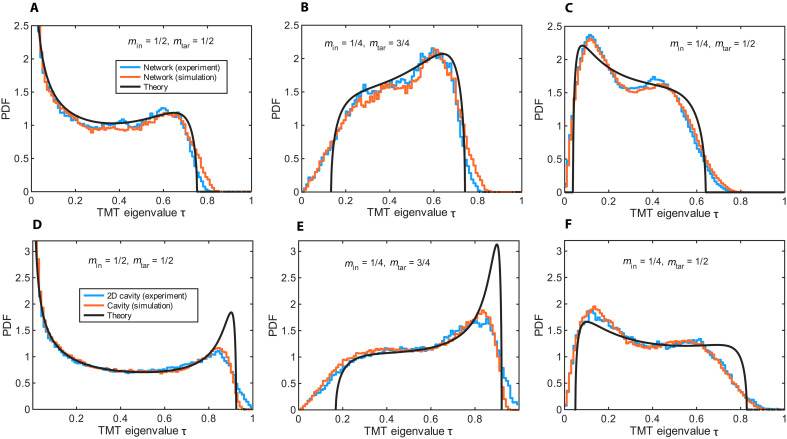
Comparison of experimental, simulated, and theoretical TMT eigenvalue distributions for complex networks and 2D cavity models. (**A** to **C**) PDF of TMT eigenvalues of a fully connected network consisting of eight “supervertices” (see Materials and Methods and fig. S1), each connected to a TL with a coupling parameter Γ ≈ 0.95. Various channel ratios $m_{\text{in}}$ and $m_{\text{tar}}$ are considered. Experimental results (blue lines) and simulations (orange lines) are shown. Intrinsic cable losses are modeled with an imaginary part of the refractive index of ≈2 × 10^−3^, corresponding to absorption *A* ≈ 0.35. The statistical analysis is performed over the frequency range [1.5 GHz, 4.5 GHz]. Predictions from the diagrammatic approach are shown (black lines), with an absorption factor a=A/(1−A/Γ)≈0.55. (**D** to **F**) The same analysis as in (A) to (C), but for the 2D cavity shown in Fig. 1B attached to eight TLs. Experimental results (blue lines) and simulations (orange lines) are presented. Statistics for the experimental data are collected over the frequency range [8 GHz, 15 GHz], whereas simulations use effective Hamiltonians ${\text{H}}_{0}$ taken from the GOE with dimensionality *N* = 100. The simulated system is characterized by a coupling parameter Γ ≈ 0.92 and absorption *A* ≈ 0.1. Predictions from the diagrammatic approach are shown (black lines), with an absorption factor a=A/(1−A/Γ)≈0.11.

Next, we compare the results of our theoretical model with experimental measurements conducted in complex networks, 2D chaotic cavities, and 3D reverberation chambers. To facilitate the comparison, all cases involved *M* = 8 channels. We first consider the microwave network consisting of 28 coaxial cables, as shown in Fig. 1A. To achieve maximum all-to-all connectivity and ensure chaotic wave dynamics, we designed eight-port “supervertices”—each consisting of a combination of six Tee junctions. Each supervertex was coupled to a TL. In the frequency range [1.5 GHz, 4.5 GHz] where the measurements were performed, the average coupling parameter was estimated as $\Gamma =1-{|\langle {\text{S}}_{\alpha \alpha }\rangle |}^{2}\approx 0.97$ (see Materials and Methods and section S1). From the measured scattering matrices, we extracted the TMT matrices *T* and evaluated their eigenvalues τ. Some typical TMT distributions P(τ) for various $m_{\text{in}}$, $m_{\text{tar}}$ configurations are shown in Fig. 3 (A to C) (blue lines). For comparison, we also report the results of simulations for the corresponding network with the same values of *M* = 8 channels, coupling Γ, and absorption *A* ≈ 0.35 as in the experiment (orange lines). The diagrammatic predictions, formally derived in the limit $M_{\text{in}},M_{\text{tar}}\gg 1$, for a=A/(1−A/Γ)≈0.55 are also shown (black lines) in the same figures. The good agreement reveals the robustness of the analytical theory even for a moderate number of channels.

The same experimental analysis was carried out for the 2D chaotic cavity shown in Fig. 1B. Statistics (blue lines in Fig. 3, D to F) were generated from scattering measurements in the frequency range [8 GHz, 15 GHz]. Simulation results for the cavity, based on random matrix modeling (see orange lines in Fig. 3), indicate that the microscopic model parameters that best fit the experimental data are $\left(\gamma ,\gamma^{\prime }\right)=\left(0.56,0.003\right)$, corresponding to the ensemble-averaged macroscopic model parameters (Γ,A)≈(0.92,0.1). These values have to be compared with the experimentally estimated parameters (Γ,A)≈(0.95,0.1). In the simulations, the ensemble average was performed over realizations of 100 × 100 random Gaussian matrices, whereas in the experiment, the ensemble was constructed over different frequencies. In the same figure, we also report the predictions of the diagrammatic approach (black lines), using a=A/(1−A/Γ)≈0.11. Overall, good agreement is observed between the experimental, numerical, and theoretical results.

A prominent feature in all cases shown in Fig. 3 is the suppression of open channels and the emergence of a statistical gap, driven by absorption in both setups and, in the last column, also by the breakdown of the CCC. The greater absorption in the network compared to the cavity is evident from the more pronounced contraction of the eigenvalue spectrum toward smaller τ values. It is also worth highlighting the asymmetric case of Fig. 3 (B and E), where the distribution P(τ) peaks near the upper bound of the TMT eigenvalues. A comparison with the analogous case in Fig. 2E reveals that the critical factor here is the increased coupling parameter Γ, which approaches perfect coupling.

When strong incomplete channel control or substantial losses arise, as in the 3D cavity shown in Fig. 1C, the correlations in the TMT matrix **T** are progressively lost (see section S3 and fig. S3). In such cases, we find that the distribution of eigenvalues normalized by their mean, P(x=τ/〈τ〉), derived using the diagrammatic approach, converges to the Marchenko-Pastur law for rectangular random matrices with uncorrelated Gaussian elements (*48*, *49*), $P\left(x\right)=\frac{\sqrt{\left(x^{+}-x\right)\left(x-x^{-}\right)}}{2\pi \left(m_{\text{in}}/m_{\text{tar}}\right)x}$, where $x^{\pm }={\left(1\pm \sqrt{m_{\text{in}}/m_{\text{tar}}}\right)}^{2}$. This result is independent of both the coupling strength Γ and absorption. In addition, as derived in section S2.D, the average transmission coefficient is given by $\langle \tau \rangle =m_{\text{tar}}\Gamma /\left(1+a/\Gamma \right)=m_{\text{tar}}\left(\Gamma -A\right)$, implying that the largest and smallest accessible transmission coefficients are $\tau^{\pm }={\left(\sqrt{m_{\text{in}}}\pm \sqrt{m_{\text{tar}}}\right)}^{2}\left(\Gamma -A\right)$, covering a range $\tau^{+}-\tau^{-}=4\sqrt{m_{\text{in}}m_{\text{tar}}}\left(\Gamma -A\right)$. These results have been confirmed by measurements in the 3D reverberation chamber (see section S3 and fig. S4). Even when the total mean absorption is as high as *A* = 83% and only 50% of the channels are detected, it is still possible to find a wavefront that achieves 25% targeted transmission.

### Extreme TMT bounds

Our diagrammatic approach takes advantage of the complex nature of scattering processes in wave-chaotic environments to derive a universal description of the dependence of the TMT eigenvalue density on intrinsic losses, coupling strength between the scattering domain and the interrogating/targeted antennas, and incomplete channel control—factors often present in realistic operational scenarios, such as indoor wireless communications.

The main findings of our analysis are as follows. For lossless systems, the bimodal eigenvalue statistics predicted for perfect and symmetric coupling (Γ = 1, $m_{\text{in}}=m_{\text{tar}}=1/2$) becomes increasingly skewed toward smaller TMT eigenvalues as the coupling parameter decreases. In scenarios where the CCC is preserved ($m_{\text{in}}+m_{\text{tar}}=1$), the TMT eigenvalue distribution extends up to unity. A statistical gap near the open channels forms only for very weak coupling Γ in asymmetric channel scenarios ($m_{\text{in}}\neq m_{\text{tar}}$). This gap widens in cases of the broken CCC, where the bimodal nature of the statistics is entirely suppressed. In the presence of losses, the statistical gap becomes even more pronounced and is observed even under the CCC. An important result of our analysis is the identification of conditions under which the TMT distribution develops a skewed bimodal shape, peaking around the maximum eigenvalues. This optimal TMT scenario occurs when Γ → 1 and the number of injected and targeted channels are unequal. We note that, because of reciprocity, all results remain valid for $m_{\text{in}}>m_{\text{tar}}$, except for the emergence of $M_{\text{in}}-M_{\text{tar}}$ zero TMT eigenvalues. These contribute an additional delta function term to the probability density, with a statistical weight of $\left(M_{\text{in}}-M_{\text{tar}}\right)/\left(M_{\text{in}}+M_{\text{tar}}\right)$.

**Fig. 4.**
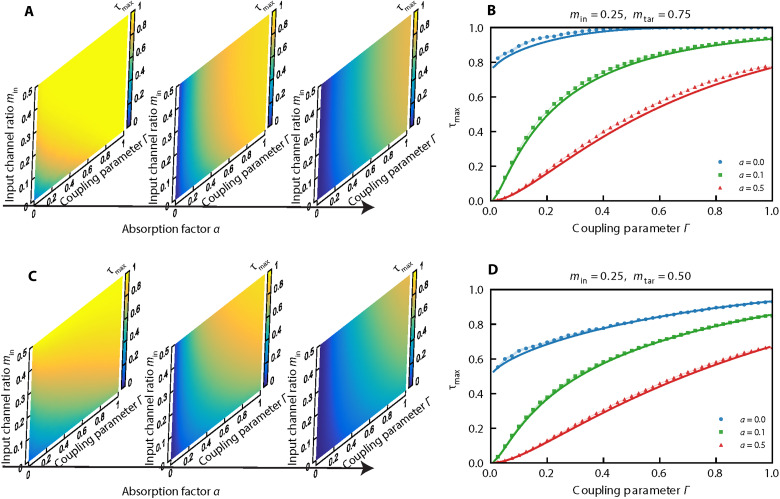
Extreme TMT bounds. (**A**) Maximum transmission τ_max_ as a function of the input channel ratio *m*_in_ and the coupling parameter Γ for a complementary channel configuration ($m_{\text{in}}+m_{\text{tar}}=1$) and for three representative absorption factors: *a* = 0, 0.1, and 0.5 (left to right). (**B**) Representative case comparing numerically extracted values of τ_max_ (points) with diagrammatic predictions for the edge of the marginal distribution in the large-*N*, *M* limit (solid lines), for $m_{\text{in}}=0.25,m_{\text{tar}}=0.75$. (**C**) Same as in (A) but for a noncomplementary case with a fixed output channel ratio $m_{\text{tar}}=0.5$. (**D**) Same as in (B) but for a noncomplementary case with $m_{\text{in}}=0.25,m_{\text{tar}}=0.5$. The simulations shown here are based on a chaotic cavity modeled by an ensemble of 1000 realizations of effective Hamiltonians ${\text{H}}_{0}$ of dimensionality *N* = 600 and *M* = 160 number of TLs.

The extreme TMT eigenvalues provide a direct estimate of the efficiency of CWS schemes. In our simulations, where finite-size matrices are used, we have identified the upper bound of the TMT eigenvalues $\tau_{\text{max}}$, via the following operational definition $\int_{0}^{\tau_{\text{max}}}P\left(\tau \right)\mathrm{d\tau}=1-\varepsilon$ where ε = 10^−3^. Figure 4 summarizes the dependence of the upper bound τ_max_ on the absorption parameter *a*, the coupling parameter Γ, and the degree of channel control determined by *m*_in_ and *m*_tar_. These extreme eigenvalues are predicted by our diagrammatic theory as the solutions of an explicit set of analytical equations (see Materials and Methods and section S2.E).

Figure 4 (A and B) illustrates the scenario of the CCC, where the maximum eigenvalue τ_max_ achieves near-perfect TMT for Γ ≈ 1, even as losses increase. In addition, symmetric channel control ($m_{\text{in}}\to m_{\text{tar}}$) enhances the perfect TMT scenario. We predict that any nonabsorbing complex cavity will exhibit reflectionless states—as studied in refs. (*8*, *9*, *13*) and defined as states with $\tau_{\text{max}}=1$ under the CCC—irrespective of the fraction $m_{\text{in}}$ of injected channels and for almost any coupling strength Γ.

Figure 4 (C and D) shows the case of noncomplementary channel configurations ($m_{\text{in}}+m_{\text{tar}}<1$, with $m_{\text{tar}}=0.5$). Here, perfect TMT is achieved when Γ → 1, but incomplete channel control ($m_{\text{in}}\to 0$) becomes detrimental even under ideal coupling (Γ = 1). Nonetheless, the theory reveals that good TMT performance, characterized by $\tau_{\text{max}}≳0.6$, remains accessible under moderate conditions: $m_{\text{in}}≳0.2$, Γ ≳ 0.5 and *a* ≲ 0.1.

## DISCUSSION

We have presented a statistical theory of TMT that captures universal features of wave control in chaotic environments dominated by multimode interference and partial channel access. Our diagrammatic approach, supported by numerical simulations and experimental validation, provides a predictive framework for understanding and optimizing energy delivery in complex systems. In particular, the theory provides closed-form expressions for the mean absorption, transmittance, and the eigenvalue distribution of TMTs for both balanced and imbalanced channel control. Also, it allows us to avoid computationally expensive matrix manipulations and their statistical processing that are typically needed for evaluating the optimal bounds. Instead, one has to solve numerically two nonlinear equations.

An important conclusion of our work is the prediction that near-optimal transmission remains feasible even under realistic constraints, including internal losses, limited coupling, and incomplete control. This means that, when probing a wave-chaotic scattering setup over a certain bandwidth Δω, there is a high probability to find TMT excitations that transmit energy from a controlled set of input channels $M_{\text{in}}$ to another set of targeted channels $M_{\text{tar}}$ with near-unity efficiency. Our theoretical analysis provides a statistical estimation of the number of such near-unity TMT excitations $∼\frac{\Delta \omega }{\xi }\int_{1-\varepsilon }^{1}P\left(\tau \right)\mathrm{d\tau}$ where ξ is the correlation bandwidth (Ericson width) that characterizes the frequency scale over which the scattering matrix remains statistically correlated.

Our framework accurately predicts the emergence of reflectionless states, statistical transmission gaps, and skewed bimodal eigenvalue distributions. The generality of these findings has been confirmed across diverse experimental platforms, including quasi-1D microwave networks, 2D chaotic cavities, and 3D reverberation chambers. This robustness suggests that the theory extends naturally beyond electromagnetic systems to optical, acoustic, and mechanical wave domains. It is important to emphasize, however, that our results apply specifically to wave-chaotic systems (that is, systems smaller than the mean free path, where ergodicity arises from complex boundaries) and are fundamentally distinct from predictions for diffusive transport, which explicitly depend on the mean free path, system size, and dimensionality.

It will be interesting to extend our analysis beyond the ergodic constraints underlying the theoretical modeling and incorporate semiclassical features (like short orbits, scars, etc.) and localization effects. Looking ahead, the statistical perspective developed here opens promising directions for controlling waves in nonlinear, time-varying, or dynamically reconfigurable media. More broadly, this work provides a practical and predictive foundation for designing wave-based technologies, from communication and imaging to energy delivery, embracing complexity rather than resisting it.

## MATERIALS AND METHODS

### Description of the experimental microwave network

The microwave network is formed by coaxial cables (bonds) with physical lengths between 10 and 50 cm that are incommensurate with one another. These cables are connected with one another via *N* = 8 supervertices. Each supervertex is characterized by its valency $v_{n}$ (n=1⋯N) indicating the number of cables that emanate from it and are connected to other supervertices of the network. In our case, all supervertices have been constructed to have *v* = 7 and were assembled using six Tee junctions (see section S1 for details). Five of these Tee junctions have one female connector, whereas the sixth Tee-junction consists of all male connectors. This setup results in a fully connected chaotic microwave network. At each supervertex, we have attached one TL supporting a single propagating mode connected to one port of a VNA, which is used to measure the scattering parameters. The 8 × 8 scattering matrix has been measured via multiple measurements using a two-port VNA. Each measurement used the two channels attached to the VNA, whereas the remaining six channels were connected to 50-ohm loads.

The network was interrogated in the frequency range [1.5 GHz, 4.5 GHz], where the 8 × 8 scattering matrix that describes the scattering process at the supervertex has matrix elements that are approximately constant. Within this range, the average coupling parameter Γ between the network and the leads is constant and takes the approximate value Γ ≈ 0.97. The high value of Γ is associated with strong internal interferences occurring when the six Tee junctions are combined together to create the supervertex (see section S1 for more details).

The experimental setup involves ohmic losses occurring at the cables. The loss of the cables is encoded in the imaginary part of its refractive index, which could be obtained via a best fitting of the measured frequency-dependent transmission [$t\left(\omega \right)=e^{\mathrm{i\omega}/c\left(n_{r}+{\text{in}}_{i}\right)L}$] through a cable of a specific length. Best fitting analysis indicated that *n*_*i*_ ≈ 2 × 10^−3^ whereas *n*_*r*_ ≈ 1.212.

### Description of the experimental microwave cavities

An image of the quasi-2D cavity of dimensions *L* = *W* = 205.74 mm and *h* = 10.16 mm is shown in Fig. 1B. The cavity is 2D as only a single vertical polarization can propagate within the frequency range of interest [8 GHz, 15 GHz]. In random positions inside the cavity, we have placed metallic cylinders that act as scatterers. The cavity is interrogated with eight antennas. We measure the 8 × 8 scattering matrix using a VNA between eight antennas that are matched coax-to-waveguide transitions attached to the cavity. A statistical ensemble of TMT eigenvalues is constructed by measuring the scattering matrix at various frequencies inside the operational frequency range with a frequency step of 0.28 MHz.

An image of the 3D chaotic enclosure (reverberation chamber) of dimensions 1.75 m by 1.5 m by 2 m is shown in Fig. 1C. The scattering matrix is measured between eight antennas that are commercial Wi-Fi antennas (ANT-24G-HL90-SMA) matched at 2.4 GHz. Two groups of four antennas are aligned and regularly spaced by 6.5 cm, which is ∼λ/2 of the central frequency. The orientation of the two groups is orthogonal to suppress direct paths. The reverberation chamber is equipped with two mechanical stirrers (a horizontal and a vertical one) that allow us to generate an ensemble of random configurations of the scattering matrix. An ensemble of 40 random configurations is obtained from the rotation of the stirrers by steps of 3°. For better statistical processing of TMT eigenvalues, we have generated, for each cavity configuration, a number of scattering matrices corresponding to different frequencies in the range [2 GHz, 3 GHz].

### Scattering theory of networks

Microwave networks, consisting of *n* = 1, …, *N* vertices, are prototype systems that have been used successfully for the study of the universal properties of wave-chaotic systems. Two vertices *n*,*m* are coupled together via coaxial cables (bonds) of length $l_{\mathrm{nm}}$. In the studies of wave-chaos, it is typically assumed that the bond lengths are incommensurate with one another (*29*). The position $x_{\mathrm{nm}}$ on a b≡(n,m) bond is defined to be $x_{\mathrm{nm}}=0$ $\left(l_{\mathrm{nm}}\right)$ on vertex *n* (*m*). The field $\psi_{b}\left(x_{\mathrm{nm}}\right)$ on each bond satisfies the Helmholtz equation

$$\frac{d^{2}}{{\mathrm{dx}}_{\mathrm{nm}}^{2}}\psi_{b}\left(x_{\mathrm{nm}}\right)+k^{2}\psi_{b}\left(x_{\mathrm{nm}}\right)=0$$ (3)

where $k=\omega n/c_{0}$ is the wave number, ω is the angular frequency, $c_{0}$ is the speed of light in vacuum, and $n=n_{r}+{\text{in}}_{i}$ is the complex-valued relative refraction index with imaginary part $n_{i}$ indicating the losses of the coaxial cables. The solution of Eq. 3 is $\psi_{b}\left(x_{nm}\right)=\phi_{n}\frac{\text{sin}k\left(l_{b}-x_{nm}\right)}{\text{sin}{\mathrm{kl}}_{b}}+\phi_{m}\frac{\text{sin}kx_{nm}}{\text{sin}{\mathrm{kl}}_{b}}$, where $\psi_{b}\left(0\right)=\phi_{n}$ and $\psi_{b}\left(l_{b}\right)=\phi_{m}$ are the values of the field at the vertices.

We turn the compact network to a scattering setup by attaching TLs α=1,⋯,M to M≤N vertices. The field at the α − th TL takes the form $\psi_{\alpha }\left(x\right)=I_{\alpha }e^{-\mathrm{ikx}}+O_{\alpha }e^{+\mathrm{ikx}}$ for x≥0 where *x* = 0 is the position of the vertex and $I_{\alpha },O_{\alpha }$ indicate the incoming and outgoing wave amplitudes at the αth TL.

The field and current continuity on each vertex *n* could be combined to give the scattering matrix Eq. 1 (*29*) where the N×N matrix M(ω) encodes the topology of the network (connectivity and length of bonds) and has elements

$${\text{M}}_{\mathrm{nm}}\left(\omega \right)=\left\{\begin{array}{cc} -\sum_{l\neq n}A_{\mathrm{nl}}\text{cot}{\mathrm{kl}}_{\mathrm{nl}}, & \text{if}\;n=m\\ A_{\mathrm{nm}}\text{csc}{\mathrm{kl}}_{\mathrm{nm}}, & \text{if}\;n\neq m \end{array}\right.$$ (4)

where the adjacency matrix $A_{\mathrm{nl}}$ takes the values 1(0) when two vertices *n*,*l* are (not) connected. Last, the *N* × *M* matrix **D** describes the connection between the TLs α=1,⋯,M and the specific vertices where the TLs are attached. It has matrix elements ${\text{D}}_{n\alpha }=1$ if the αth TL is attached to vertex *n* and zero otherwise.

### Analytical solutions for the eigenvalue probability density function

The theoretical curves for the TMT eigenvalue distributions in Figs. 2 and 3 have been obtained by solving the equations $F_{\text{in}}\left(z\right)=0$ and $F_{\text{tar}}\left(z\right)=0$ for the self-energy components $\Sigma_{\text{in}}$ and $\Sigma_{\text{tar}}$, where

$$\begin{array}{c} F_{\text{in}}\left(z\right)=\frac{\Sigma_{\text{in}}}{1-\Sigma_{\text{in}}\Sigma_{\text{tar}}}-\frac{a\Sigma_{\text{in}}}{{\left(1-\Sigma_{\text{in}}\Sigma_{\text{tar}}\right)}^{2}}\\ -\frac{1-\Gamma }{1-\left(1-\Gamma \right)\Sigma_{\text{in}}\Sigma_{\text{tar}}}\left[\left(1+\frac{\alpha m_{\text{in}}}{1-\alpha }+\frac{\beta m_{\text{tar}}}{1-\beta }\right)\Sigma_{\text{in}}+\frac{\Gamma }{\sqrt{z}\left(1-\Gamma \right)}\frac{m_{\text{in}}}{1-\beta }\right] \end{array}$$ (5)

$$\begin{array}{c} F_{\text{tar}}\left(z\right)=\frac{\Sigma_{\text{tar}}}{1-\Sigma_{\text{in}}\Sigma_{\text{tar}}}-\frac{a\Sigma_{\text{tar}}}{{\left(1-\Sigma_{\text{in}}\Sigma_{\text{tar}}\right)}^{2}}\\ -\frac{1-\Gamma }{1-\left(1-\Gamma \right)\Sigma_{\text{in}}\Sigma_{\text{tar}}}\left[\left(1+\frac{\alpha m_{\text{in}}}{1-\alpha }+\frac{\beta m_{\text{tar}}}{1-\beta }\right)\Sigma_{\text{tar}}+\frac{\Gamma }{\sqrt{z}\left(1-\Gamma \right)}\frac{m_{\text{tar}}}{1-\beta }\right] \end{array}$$ (6)

with $\alpha =\frac{{\Gamma \Sigma }_{\text{tar}}/\sqrt{z}}{1-\left(1-\Gamma \right)\Sigma_{\text{in}}\Sigma_{\text{tar}}}$, $\beta =\frac{{\Gamma \Sigma }_{\text{in}}/\sqrt{z}}{1-\left(1-\Gamma \right)\Sigma_{\text{in}}\Sigma_{\text{tar}}}$, and inserting their values into Eq. 2.

In addition, the upper bound $\tau_{\text{max}}$ shown in Fig. 4 can be obtained by solving the system of equations composed of $F_{\text{in}}\left(\tau_{\text{max}}\right)=0$, $F_{\text{tar}}\left(\tau_{\text{max}}\right)=0$, and $\partial_{\Sigma_{\text{in}}}F_{\text{in}}\left(\tau_{\text{max}}\right)\partial_{\Sigma_{\mathrm{tar}}}F_{\text{tar}}\left(\tau_{\text{max}}\right)=\partial_{\Sigma_{\mathrm{tar}}}F_{\text{in}}\left(\tau_{\text{max}}\right)\partial_{\Sigma_{\text{in}}}F_{\text{tar}}\left(\tau_{\text{max}}\right)$. These results are demonstrated in section S2.

## Supplementary Materials

### This PDF file includes:

Sections S1 to S3 Figs. S1 to S4 References
