# Supplementary material for: Guiding waves through chaos: Universal bounds for targeted mode transport
Source: Sci Adv. 2026 Jan 28;12(5):eaeb1158. doi: 10.1126/sciadv.aeb1158 (PMC12851031; doi:10.1126/sciadv.aeb1158)
Supplement: Supplementary file 1 — Sections S1 to S3 Figs. S1 to S4 References [file sciadv.aeb1158_sm.pdf]

Science Advances
AAAS

# Supplementary Materials for

## Guiding waves through chaos: Universal bounds for targeted mode transport

Cheng-Zhen Wang *et al.*

Corresponding author: Arthur Goetschy, arthur.goetschy@espci.psl.eu; Tsampikos Kottos, tkottos@wesleyan.edu

*Sci. Adv.* **12**, eaeb1158 (2026)
DOI: 10.1126/sciadv.aeb1158

**This PDF file includes:**

Sections S1 to S3
Figs. S1 to S4
References

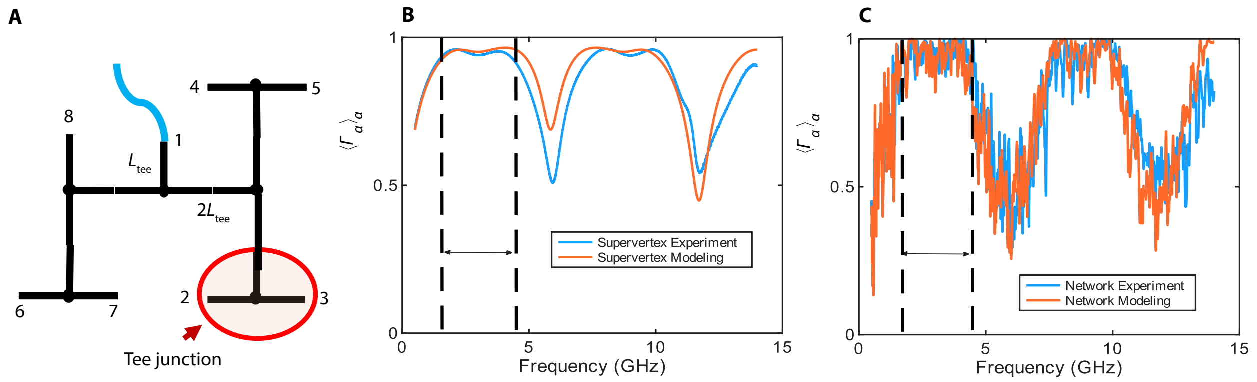


**Figure S1**: **Design and coupling parameter characteristics of an eight-port supervertex. (A)** Schematics of the eight-port supervertex consisting of six Tee-junctions coupled together as indicated in the figure. The TL (blue line) is indicatively coupled to one of the eight ports of the supervertex. **(B)** Coupling parameter $\langle\Gamma_\alpha\rangle_\alpha$ versus frequency in a broad frequency range for a single supervertex. Blue line corresponds to the experimental data (averaged over the eight channels) while the orange line is the result of our theoretical modeling. **(C)** The same as in **(B)** but now for the case of a network consisting of eight supervertices that are all coupled together.

# S1 Experimental characterization and theoretical modeling of the supervertices in networks

The scattering process at each vertex of the network is characterized by a scattering matrix $\sigma$ which, in the absence of losses, is unitary. When a vertex is considered point-like, the implementation of field continuity and current conservation leads to the so-called Neumann vertex scattering matrix $\sigma_{nm}^{(N)} = -\delta_{nm} + \frac{2}{v_n}$ where $v_n$ is the valency of the $n$−th vertex, indicating the number $m$ of other vertices of the network that are connected with it (*29*). In case that this vertex is coupled to a TL the above expression has to be modified by changing $v_n \rightarrow v_n + 1$ in order to take into account the TL. An experimental realization of a Neumann vertex is provided by standard three-prong Tee-junctions where $v = 3$ in the absence of TL, and $v = 2$ when a TL is also attached to the vertex.

The simplicity of the vertex Neumann scattering matrix allows us to evaluate immediately the coupling parameter which takes the frequency independent form $\Gamma_\alpha \equiv 1 - |\langle\sigma_{\alpha,\alpha}^{(N)}\rangle|^2 = 1 - \left(1 - \frac{2}{v_\alpha+1}\right)^2$.

In the case of super-vertices, however, the vertex scattering matrix is not (necessarily) modeled

by a Neumann matrix. Instead, internal wave interferences occurring among the various Tee-junction components that form the super-vertex, lead to a rapidly fluctuating vertex scattering matrix with respect to frequency sweeping. As a result, the $\Gamma$ parameter has also strong frequency dependence. Additionally, losses in the super-junction might further complicate the scattering process.

We have modeled this behavior theoretically by explicitly incorporating in the supervertex scattering modeling, six Tee-junctions coupled together to create an eight-port supervertex, see Fig. S1(**A**). Internal losses within the supervertex has been modeled by an imaginary part in the refractive index, $n_i \approx 2 \times 10^{-3}$. In Fig. S1(**B**) we report the results of our modeling for the coupling parameter $\langle \Gamma_\alpha \rangle_\alpha$ (orange line) against the measured coupling parameter for the eight-port supervertex (blue line). The averaging $\langle \cdots \rangle_\alpha$ is among the eight channels of the supervertex and the results are plotted versus frequency. The good agreement between theory and experiment indicate that our modeling captures successfully the scattering process at the supervertices.

The results reported in Fig. S1(**B**) indicate a strong frequency dependence of the coupling parameter. At the same time, we identify frequency domains where $\langle \Gamma_\alpha \rangle_\alpha$ is approximately constant. The same overall behavior of $\langle \Gamma_\alpha \rangle_\alpha$ versus frequency, appears also for the case of the fully connected network of eight supervertices, see Fig. S1(**C**). We have, therefore, chosen for the analysis of the probability distribution of the TMT eigenvalues, the frequency range [1.5 GHz, 4.5 GHz] where the coupling parameter is approximately constant with a mean value $\Gamma \approx 0.97$.

## S2 Theoretical predictions

### S2.A Definition of the model

Consider a lossless scattering setup, such as a chaotic cavity, involving $M$ physical channels characterized by a coupling parameter $\Gamma$. The transport properties of such a system are described by an $M$-dimensional scattering matrix $S$. When inherent (Ohmic) losses are introduced, the transport can instead be described by an effective scattering matrix $S_{\text{eff}}$ of enlarged dimension $\tilde{M} = M + M'$. In this framework, the additional $M' \gg M$ channels account for the Ohmic losses and are characterized by a coupling parameter $\Gamma' \ll \Gamma$ (*50, 51*).

Furthermore, assuming all channels of the complex system are equivalent except for their coupling, we model the total system as comprising a perfectly coupled and lossless subsystem

associated with a $\tilde{M} \times \tilde{M}$ unitary matrix $S_0$, attached to a barrier that encapsulates the effects of imperfect coupling. The $2\tilde{M} \times 2\tilde{M}$ scattering matrix of the barrier is expressed as:

$$S_1 = \begin{pmatrix} r & t' \\ t & r' \end{pmatrix}, \tag{S1}$$

Without loss of generality, we express the transmission and reflection matrices of the barrier in a basis where they are diagonal. These matrices are given by:

$$t = t' = \begin{pmatrix} \sqrt{\Gamma}\mathbb{1}_M & 0 \\ 0 & \sqrt{\Gamma'}\mathbb{1}_{M'} \end{pmatrix}, \tag{S2}$$

and

$$r = -r' = \begin{pmatrix} \sqrt{1-\Gamma}\mathbb{1}_M & 0 \\ 0 & \sqrt{1-\Gamma'}\mathbb{1}_{M'} \end{pmatrix}. \tag{S3}$$

By summing all scattering sequences contributing to the interaction between the system's barrier and the perfectly coupled subsystem, we obtain the following expression for the total scattering matrix:

$$\begin{aligned} S_{\text{eff}} &= r + t'S_0t + t'S_0r'S_0t + t'(S_0r')^2S_0t + \ldots \\ &= r + t'\frac{1}{\mathbb{1}_{\tilde{M}} - S_0r'}S_0t. \end{aligned} \tag{S4}$$

Using the projectors $P_{\text{in}} = \mathbb{1}_{M_{\text{in}}}$ and $P_{\text{tar}} = \mathbb{1}_{M_{\text{tar}}}$, which act on the $M$-dimensional (physical) channel subspace with $M_{\text{in}}, M_{\text{tar}} \leq M$, and satisfy the orthogonality constraint $P_{\text{in}} \cdot P_{\text{tar}} = 0$, we obtain the $M_{\text{tar}} \times M_{\text{in}}$ scattering submatrix $\tilde{S}$:

$$\tilde{S} = P_{\text{tar}}S_{\text{eff}}P_{\text{in}} = P_{\text{tar}}t'\frac{1}{1 - S_0r'}S_0tP_{\text{in}}, \tag{S5}$$

from which we evaluate the $M_{\text{in}} \times M_{\text{in}}$ Hermitian TMT matrix $T = \tilde{S}^\dagger\tilde{S}$. In the following, we will focus on the evaluation of the resolvent:

$$g_T(z) = \frac{1}{M_{\text{in}}}\left\langle \text{Tr}\frac{1}{z - T} \right\rangle, \tag{S6}$$

which provides access to the density of eigenvalues $\tau$ of the matrix $T$ via $\mathcal{P}(\tau) = -\frac{1}{\pi}\lim_{\eta\to 0^+}\text{Im}[g_T(\tau + i\eta)]$.

### S2.B A special case: perfect coupling and no absorption

In the case of perfect coupling ($\Gamma = 1$) and no absorption ($\Gamma' = 0$), the filtered scattering matrix can be expressed as $\tilde{S} = P_{\text{tar}} S_0 P_{\text{in}}$, where $S_0$ is an $M \times M$ matrix uniformly distributed within the unitary group. Since all scattering channels of the matrix to be filtered are statistically equivalent in this scenario, we can use the filtered random matrix (FRM) theory (*21*), which provides $g_T(z)$ as the solution of the implicit equation:

$$N(z) g_{S_0^\dagger S_0} \left[ \frac{N(z)^2}{D(z)} \right] = D(z). \tag{S7}$$

Here, $N(z)$ and $D(z)$ are auxiliary functions defined as:

$$N(z) = z\, m_{\text{in}} g_T(z) + 1 - m_{\text{in}}, \tag{S8}$$

$$D(z) = m_{\text{in}} g_T(z) \left[ z m_{\text{in}} g_T(z) + m_{\text{tar}} - m_{\text{in}} \right], \tag{S9}$$

with $m_{\text{in}} = M_{\text{in}}/M$ and $m_{\text{tar}} = M_{\text{tar}}/M$. Since $S_0^\dagger S_0 = \mathbb{1}_M$, its resolvent has a simple form: $g_{S_0^\dagger S_0}(z) = 1/(z-1)$. The solution of Eq. (S7) is given by:

$$g_T(z) = \frac{\left[ m_{\text{in}} - m_{\text{tar}} + (1 - 2m_{\text{in}}) z - \sqrt{(m_{\text{in}} - m_{\text{tar}})^2 + z(z - 2m_{\text{in}} - 2m_{\text{tar}} + 4 m_{\text{in}} m_{\text{tar}})} \right]}{2 m_{\text{in}} z (1 - z)}, \tag{S10}$$

from which we deduce the eigenvalue density:

$$\mathcal{P}(\tau) = \frac{1}{\pi} \frac{\sqrt{(\tau^+ - \tau)(\tau - \tau^-)}}{2 m_{\text{in}} \tau (1 - \tau)} + \max\left(1 - \frac{m_{\text{tar}}}{m_{\text{in}}}, 0\right) \delta(\tau), \tag{S11}$$

where the bounds $\tau^\pm$ are given by:

$$\tau^\pm = m_{\text{in}} + m_{\text{tar}} - 2 m_{\text{in}} m_{\text{tar}} \pm 2\sqrt{m_{\text{in}} m_{\text{tar}} (1 - m_{\text{in}})(1 - m_{\text{tar}})}. \tag{S12}$$

We note, in particular, that under the complementary channel condition (CCC) $m_{\text{in}} + m_{\text{tar}} = 1$, the upper bound simplifies to $\tau^+ = 1$ for all $m_{\text{in}}$. This indicates a non-zero probability of finding reflectionless states in the system. On the other hand, the lower bound $\tau^- = (1 - 2m_{\text{in}})^2$ is never zero except in the symmetric case $m_{\text{in}} = m_{\text{tar}} = 1/2$.

### S2.C Self-consistent equations in the general case

To evaluate Eq. (S6), we use a diagrammatic approach that takes advantages of the fact that $S_0$ is uniformly distributed within the unitary group (*52*). However, a direct series expansion of Eq. (S6)

in powers of $S_0$ is not suitable. A more convenient approach is to work in a duplicated space and introduce

$$\hat{S} = \begin{pmatrix} S^\dagger & 0 \\ 0 & S \end{pmatrix}, \quad \hat{P} = \begin{pmatrix} 0 & P_{\rm tar} \\ P_{\rm in} & 0 \end{pmatrix}, \tag{S13}$$

as well as the matrix resolvent

$$\hat{G}(z) = \begin{pmatrix} 0 & \hat{G}_{\rm tar} \\ \hat{G}_{\rm in} & 0 \end{pmatrix}, \tag{S14}$$

where $\hat{G}_{\rm in}(z) = \left\langle \frac{1}{z - \tilde{S}^\dagger \tilde{S}} \right\rangle$ and $\hat{G}_{\rm tar}(z) = \left\langle \frac{1}{z - \tilde{S}\tilde{S}^\dagger} \right\rangle$. Thus, $g_T(z) = {\rm Tr}[\hat{G}_{\rm in}(z)]/M_{\rm in}$. The matrix resolvent can then be written as

$$\hat{G}(z) = \left\langle \frac{1}{z - (\hat{P}\hat{S})^2} \hat{P} \right\rangle = \frac{1}{2\sqrt{z}} \sum_{\pm} \left\langle \frac{1}{\sqrt{z} \pm \hat{P}\hat{S}} \hat{P} \right\rangle. \tag{S15}$$

Noting that $\hat{P}\hat{S}\hat{P} = \hat{P}\hat{t} \left[ \mathbb{1}_{2\tilde{M}} - \hat{S}_0 \hat{r}' \right]^{-1} \hat{S}_0 \hat{t} \hat{P}$, where the following matrices have been introduced:

$$\hat{S}_0 = \begin{pmatrix} S_0^\dagger & 0 \\ 0 & S_0 \end{pmatrix}, \; \hat{t} = \begin{pmatrix} t^\dagger & 0 \\ 0 & t \end{pmatrix}, \; \hat{r}' = \begin{pmatrix} r'^\dagger & 0 \\ 0 & r' \end{pmatrix}, \tag{S16}$$

simple algebraic manipulation allows the matrix resolvent to be expressed as

$$\hat{G}(z) = \frac{\hat{P}}{2z} \sum_{\pm} \left[ \mathbb{1}_{2\tilde{M}} \pm \frac{1}{\sqrt{z}} \hat{t} (\hat{X}^\pm)^{-1} (\hat{G}^\pm - \hat{X}^\pm)(\hat{X}^\pm)^{-1} \hat{t} \hat{P} \right], \tag{S17}$$

where $\hat{X}^\pm = \hat{r}' \pm \hat{t}\hat{P}\hat{t}/\sqrt{z}$ and

$$\hat{G}^\pm = \left\langle \frac{1}{(\hat{X}^\pm)^{-1} - \hat{S}_0} \right\rangle = \frac{1}{(\hat{X}^\pm)^{-1} - \hat{\Sigma}^\pm}. \tag{S18}$$

Hence, the diagrammatic evaluation of $\hat{G}_{\rm in}(z)$ has been reduced to the evaluation of $\hat{G}^\pm$, which is technically less involved.

By combining the explicit expression of the matrix propagator, $\hat{X}^\pm = -\sqrt{\mathbb{1}_{2\tilde{M}} - \hat{\Gamma}} \pm \hat{\Gamma}\hat{P}/\sqrt{z}$, where $\hat{\Gamma} = {\rm diag}(\Gamma \mathbb{1}_M, \Gamma' \mathbb{1}_{M'}, \Gamma \mathbb{1}_M, \Gamma' \mathbb{1}_{M'})$, with the fact – demonstrated below – that the self-energy introduced in Eq. (S18) has the following simple form:

$$\hat{\Sigma}^\pm = \begin{pmatrix} 0 & \Sigma^\pm_{\rm tar} \mathbb{1}_{\tilde{M}} \\ \Sigma^\pm_{\rm in} \mathbb{1}_{\tilde{M}} & 0 \end{pmatrix}. \tag{S19}$$

We find that the resolvent we are seeking can be expressed in terms of the self-energy elements as

$$g_T(z) = \frac{1}{z}\frac{1-(1-\Gamma)\Sigma^+_{\rm in}\Sigma^+_{\rm tar}}{1-(1-\Gamma)\Sigma^+_{\rm in}\Sigma^+_{\rm tar}-\Gamma\Sigma^+_{\rm tar}/\sqrt{z}}. \tag{S20}$$

To derive this expression, we used the orthogonality condition $P_{\rm in}\cdot P_{\rm tar} = 0$ and the relation $\hat{\Sigma}^- = -\hat{\Sigma}^+$. Note that the superscripts in $\Sigma^+_{\rm in}$ and $\Sigma^+_{\rm tar}$ are omitted in Eq. (2) of the main text for simplicity of notation.

Finally, we evaluate the self-energy matrix $\hat{\Sigma}^\pm$ defined in Eq. (S18) using a diagrammatic approach. By definition, its elements include all irreducible diagrams in the expansion of $(\hat{X}^\pm)^{-1}\hat{G}^\pm(\hat{X}^\pm)^{-1} = \hat{S}_0 + \hat{S}_0\hat{X}^\pm\hat{S}_0 + \dots$. The average of the product of $\hat{S}_0$ matrix elements is given by (*52, 53*)

$$\overline{(S_{0,a_1b_1}\dots S_{0,a_mb_m})(S^*_{0,\alpha_1\beta_1}\dots S^*_{0,\alpha_m\beta_m})} = \sum_{P,P'} V_{PP'}\prod_{j=1}^{m}\delta_{a_j\alpha_{P(j)}}\delta_{b_j\beta_{P'(j)}}, \tag{S21}$$

where $P, P'$ are permutations of $\{1,\dots,m\}$, and the weights $V_{PP'}$ depend only on the cycle structure of $P^{-1}P'$: $V_{PP'} = V_{c_1\dots c_k}$, with $c_j$ the lengths of disjoint cyclic permutations in $P^{-1}P'$ ($\sum_{j=1}^{k} c_j = m$). In the limit $\tilde{M} \gg 1$, it is known that (*52*)

$$V_{c_1\dots c_k} = \prod_{j=1}^{k} V_{c_j}, \ \text{with } V_n = \frac{(-1)^{n-1}(2n-2)!}{n(n-1)!^2}\frac{1}{\tilde{M}^{2n-1}}. \tag{S22}$$

Among all possible contractions in Eq. (S21), only cycles corresponding to planar diagrams contribute substantially to the self-energy for $M_{\rm in}, M_{\rm tar} \gg 1$. These diagrams correspond to non-disjoint cyclic permutations, with $V_{PP'} \simeq V_n$. Summing over all planar diagrams, we find

$$\Sigma^\pm_{\rm in} = V_1{\rm Tr}\hat{X}^\pm_{21} + V_2({\rm Tr}\hat{X}^\pm_{21})^2{\rm Tr}\hat{X}^\pm_{12} + \dots = \sum_{n=1}^{\infty} V_n({\rm Tr}\hat{G}^\pm_{\rm in})^n({\rm Tr}\hat{G}^\pm_{\rm tar})^{n-1} = {\rm Tr}\hat{G}^\pm_{\rm in}\, H\left({\rm Tr}\hat{G}^\pm_{\rm in}{\rm Tr}\hat{G}^\pm_{\rm tar}\right), \tag{S23}$$

where $\hat{G}^\pm_{\rm in}$ and $\hat{G}^\pm_{\rm tar}$ are the non-diagonal blocks of $\hat{G}^\pm$, and

$$H(z) = \frac{1}{\tilde{M}}\sum_{n=0}^{\infty}\left(\frac{-z}{\tilde{M}^2}\right)^n\frac{(2n)!}{n!(n+1)!} = \frac{\sqrt{\tilde{M}^2+4z}-\tilde{M}}{2z} \tag{S24}$$

is the generating function of the Catalan numbers. Similarly, we find

$$\Sigma^\pm_{\rm tar} = {\rm Tr}\hat{G}^\pm_{\rm tar}\, H\left({\rm Tr}\hat{G}^\pm_{\rm in}{\rm Tr}\hat{G}^\pm_{\rm tar}\right). \tag{S25}$$

Since $\hat{S}_0$ contains only diagonal blocks, the diagonal blocks of $\hat{\Sigma}^\pm$ arise from non-planar diagrams, which can be neglected for $M_{\rm in}, M_{\rm tar} \gg 1$: $\hat{\Sigma}^\pm_{11} \simeq \hat{\Sigma}^\pm_{22} \simeq 0$.

By inverting the relations (S23) and (S25), we express $\mathrm{Tr}\hat{G}^{\pm}_{\mathrm{in}}$ and $\mathrm{Tr}\hat{G}^{\pm}_{\mathrm{tar}}$ in terms of the self-energy elements:

$$\mathrm{Tr}\hat{G}^{\pm}_{\mathrm{in}} = \frac{\tilde{M}\Sigma^{\pm}_{\mathrm{in}}}{1-\Sigma^{\pm}_{\mathrm{in}}\Sigma^{\pm}_{\mathrm{tar}}}; \quad \mathrm{Tr}\hat{G}^{\pm}_{\mathrm{tar}} = \frac{\tilde{M}\Sigma^{\pm}_{\mathrm{tar}}}{1-\Sigma^{\pm}_{\mathrm{in}}\Sigma^{\pm}_{\mathrm{tar}}}. \tag{S26}$$

Equating these expressions to the traces of the diagonal blocks of Eq. (S18), we find that the self-energy terms satisfy two coupled non-linear equations:

$$F^{\pm}_{\mathrm{in}}(z) = 0 \quad \text{and} \quad F^{\pm}_{\mathrm{tar}}(z) = 0, \tag{S27}$$

defined as

$$\begin{aligned} F^{\pm}_{\mathrm{in}}(z) &= \frac{\Sigma^{\pm}_{\mathrm{in}}}{1-\Sigma^{\pm}_{\mathrm{in}}\Sigma^{\pm}_{\mathrm{tar}}} - \frac{a\Sigma^{\pm}_{\mathrm{in}}}{(1-\Sigma^{\pm}_{\mathrm{in}}\Sigma^{\pm}_{\mathrm{tar}})^2} \\ &- \frac{1-\Gamma}{1-(1-\Gamma)\Sigma^{\pm}_{\mathrm{in}}\Sigma^{\pm}_{\mathrm{tar}}}\left[\left(1+\frac{\alpha_{\pm}m_{\mathrm{in}}}{1-\alpha_{\pm}}+\frac{\beta_{\pm}m_{\mathrm{tar}}}{1-\beta_{\pm}}\right)\Sigma^{\pm}_{\mathrm{in}} \pm \frac{\Gamma}{\sqrt{z}(1-\Gamma)}\frac{m_{\mathrm{in}}}{1-\beta_{\pm}}\right], \end{aligned} \tag{S28}$$

$$\begin{aligned} F^{\pm}_{\mathrm{tar}}(z) &= \frac{\Sigma^{\pm}_{\mathrm{tar}}}{1-\Sigma^{\pm}_{\mathrm{in}}\Sigma^{\pm}_{\mathrm{tar}}} - \frac{a\Sigma^{\pm}_{\mathrm{tar}}}{(1-\Sigma^{\pm}_{\mathrm{in}}\Sigma^{\pm}_{\mathrm{tar}})^2} \\ &- \frac{1-\Gamma}{1-(1-\Gamma)\Sigma^{\pm}_{\mathrm{in}}\Sigma^{\pm}_{\mathrm{tar}}}\left[\left(1+\frac{\alpha_{\pm}m_{\mathrm{in}}}{1-\alpha_{\pm}}+\frac{\beta_{\pm}m_{\mathrm{tar}}}{1-\beta_{\pm}}\right)\Sigma^{\pm}_{\mathrm{tar}} \pm \frac{\Gamma}{\sqrt{z}(1-\Gamma)}\frac{m_{\mathrm{tar}}}{1-\beta_{\pm}}\right], \end{aligned} \tag{S29}$$

where $\alpha_{\pm}$ and $\beta_{\pm}$ are auxiliary functions:

$$\alpha_{\pm} = \frac{\pm\Gamma\Sigma^{\pm}_{\mathrm{tar}}/\sqrt{z}}{1-(1-\Gamma)\Sigma^{\pm}_{\mathrm{in}}\Sigma^{\pm}_{\mathrm{tar}}}; \quad \beta_{\pm} = \frac{\pm\Gamma\Sigma^{\pm}_{\mathrm{in}}/\sqrt{z}}{1-(1-\Gamma)\Sigma^{\pm}_{\mathrm{in}}\Sigma^{\pm}_{\mathrm{tar}}}, \tag{S30}$$

and $a$ is the macroscopic absorption parameter in the limit $\Gamma' \to 0$ and $M' \to \infty$, with $M'\Gamma'/M$ finite:

$$a = \frac{M'\Gamma'}{M}\frac{1-\Sigma^{\pm}_{\mathrm{in}}\Sigma^{\pm}_{\mathrm{tar}}}{1-(1-\Gamma')\Sigma^{\pm}_{\mathrm{in}}\Sigma^{\pm}_{\mathrm{tar}}} \xrightarrow[\Gamma'\to 0]{M'\to\infty} \frac{M'\Gamma'}{M}. \tag{S31}$$

In the case of a lossy cavity supporting a very large number $N$ of modes and characterized by the loss rate $\gamma'$, we have $M' \simeq N$ and $\Gamma' = 1 - \frac{(1-\gamma')^2}{(1+\gamma')^2} \simeq 4\gamma'$ ($\gamma' \ll 1$), so that the absorption parameter can also be expressed as $a = 4(N/M)\gamma'$. As a result, the $M'$ channels do not introduce any additional fluctuations in the scattering properties of the cavity. The resolvent of the TMT operator is obtained by substituting the solutions $\Sigma^{+}_{\mathrm{in}}$ and $\Sigma^{+}_{\mathrm{tar}}$ from Eq. (S27) into Eq. (S20). Note that the superscripts in $F^{+}_{\mathrm{in}}$ and $F^{+}_{\mathrm{tar}}$ are omitted in the Methods section of the main text for simplicity of notation.

We point out that Eq. (S27) has explicit analytical solutions only in some special cases. When there is no absorption ($a = 0$) and the coupling is optimal ($\Gamma = 1$), we find

$$\Sigma_{\text{in}}^{\pm} = \pm\frac{-\sqrt{z}(z+m_{\text{in}}-m_{\text{tar}})+\sqrt{f(z,m_{\text{in}},m_{\text{tar}})}}{2(m_{\text{tar}}-1)z}, \tag{S32}$$

$$\Sigma_{\text{tar}}^{\pm} = \pm\frac{-\sqrt{z}(z+m_{\text{tar}}-m_{\text{in}})+\sqrt{f(z,m_{\text{in}},m_{\text{tar}})}}{2(m_{\text{in}}-1)z}, \tag{S33}$$

where $f(z, m_{\text{in}}, m_{\text{tar}}) = z^3+2(2m_{\text{in}}m_{\text{tar}}-m_{\text{in}}-m_{\text{tar}})z^2+(m_{\text{in}}-m_{\text{tar}})^2 z$. Combining these expressions with Eq. (S20), we recover the solution (S10). On the other hand, when $\Gamma \neq 1$, an explicit solution can still be found in the special case of balanced CCC ($m_{\text{in}} = m_{\text{tar}} = 1/2$) without absorption. Then, the self-energy terms are given by $\Sigma_{\text{in}}^{+} = \Sigma_{\text{tar}}^{+} = \sqrt{z} - \sqrt{z-1}$, for all $\Gamma$. The resolvent (S20) becomes

$$g_T(z) = \frac{1 + (1-\Gamma)\left[\sqrt{z(z-1)} - z\right] - \Gamma/2}{z(1-\Gamma)\left[1 + \sqrt{z(z-1)} - z\right] + \sqrt{z(z-1)}\Gamma/2}, \tag{S34}$$

which gives the bimodal eigenvalue density

$$\mathcal{P}(\tau) = \frac{1}{\pi}\frac{\Gamma(2-\Gamma)}{\sqrt{\tau(1-\tau)}\,(\Gamma^2 - 4\Gamma\tau + 4\tau)}, \tag{S35}$$

in agreement with the results of Ref. (*52*).

### S2.D Mean transmittance and mean absorption

The mean TMT eigenvalue can be derived from the distribution $\mathcal{P}(\tau)$. However, extracting its analytical expression directly from the self-consistent equations is tedious. Instead, we compute the mean directly using

$$\begin{aligned}\langle\tau\rangle = \frac{1}{M_{\text{in}}}\left\langle \text{Tr}\left(\tilde{S}^\dagger\tilde{S}\right)\right\rangle &= \frac{1}{M_{\text{in}}}\left\langle \text{Tr}\left(P_{\text{in}}t^\dagger S_0^\dagger[1 - r'^\dagger S_0^\dagger]^{-1} t'^\dagger P_{\text{tar}} t'[1 - S_0 r']^{-1} S_0 t P_{\text{in}}\right)\right\rangle \\ &= \frac{1}{M_{\text{in}}}\sum_{n=0}^{\infty}\left\langle \text{Tr}\left(P_{\text{in}}t^\dagger S_0^\dagger (r'^\dagger S_0^\dagger)^n t'^\dagger P_{\text{tar}} t'(S_0 r')^n S_0 t P_{\text{in}}\right)\right\rangle.\end{aligned} \tag{S36}$$

In the last equality, we used the fact that a product involving random unitary matrices $S_0$ and $S_0^\dagger$ is non-zero only if it contains the same number of $S_0$ and $S_0^\dagger$ matrices. Using Eq. (S21), the leading planar diagrams in Eq. (S36) are rainbow diagrams of weight $V_1^{n+1} = 1/\tilde{M}^{n+1}$. This result is identical to what would be obtained when treating $S_0$ as a Gaussian random matrix. Equation (S36) then simplifies to

$$\langle\tau\rangle = \frac{\text{Tr}(tP_{\text{in}}t^\dagger)\text{Tr}(t'^\dagger P_{\text{tar}} t')}{M_{\text{in}}\tilde{M}}\sum_{n=0}^{\infty}\left[\frac{\text{Tr}(r'^\dagger r')}{\tilde{M}}\right]^n = \frac{\Gamma^2 M_{\text{tar}}}{\tilde{M} - \text{Tr}(r'^\dagger r')} = \frac{m_{\text{tar}}\Gamma}{1 + a/\Gamma}, \tag{S37}$$

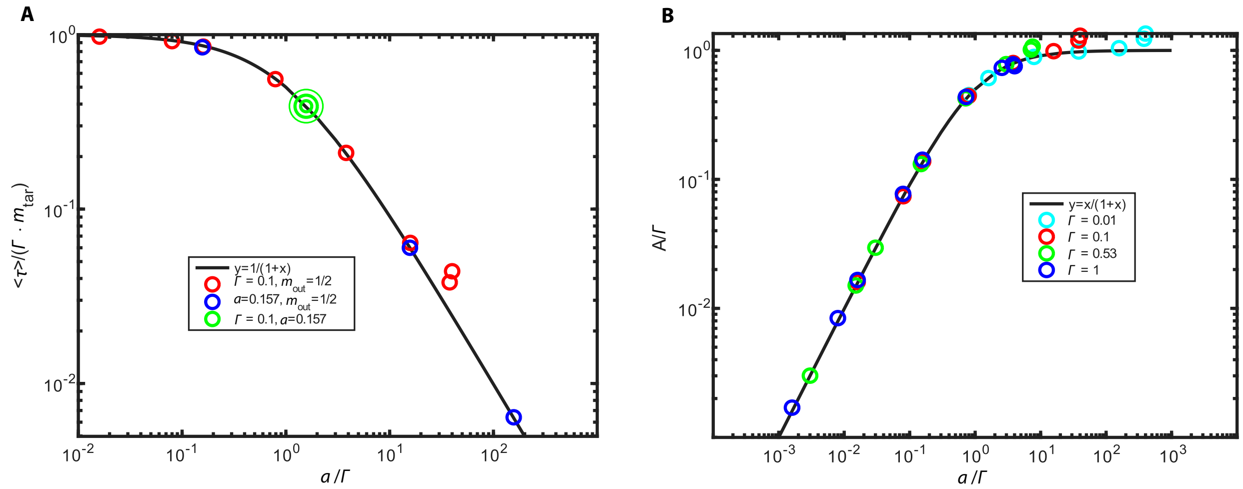


**Figure S2**: **Scaling of mean TMT eigenvalues and absorption versus the absorption factor. (A)** Rescaled mean TMT eigenvalue $\langle\tau\rangle/m_{\text{tar}}\Gamma$ versus the rescaled absorption factor $a/\Gamma$ for various combinations of $\Gamma$, $a$, and $m_{\text{tar}}$ parameters. The legend indicates the parameters kept constant while varying $a$ (red circles), $\Gamma$ (blue circles), and $m_{\text{tar}}$ (green circles of varying sizes, with smaller to larger circles corresponding to $M_{\text{in}} = 10, 10, 30$ and $M_{\text{tar}} = 40, 70, 50$, respectively). The black line represents the theoretical prediction $\langle\tau\rangle/m_{\text{tar}}\Gamma = \frac{1}{1+a/\Gamma}$. **(B)** Relationship between total absorption $A$ and the absorption factor $a$ for different coupling parameters $\Gamma$ (indicated by circles of different colors). All curves collapse onto a single universal curve when the $(x, y)$ axes are rescaled as $(a/\Gamma, A/\Gamma)$. The black line represents the theoretical prediction $A/\Gamma = \frac{a/\Gamma}{1+a/\Gamma}$. In both subfigures, the symbols correspond to results of cavity simulations using a random matrix theory model for matrices with system size $N = 300$ and $M = 80$ channels.

where we used $\mathrm{Tr}(r'^{\dagger}r') = M(1-\Gamma) + M'(1-\Gamma')$.

Similarly, the mean total absorption in the system is given by

$$A = 1 - \frac{1}{M}\left\langle \mathrm{Tr}\left(S^{\dagger}S\right)\right\rangle = 1 - \frac{1}{M}\left\langle \mathrm{Tr}\left(PS_{\mathrm{eff}}^{\dagger}PS_{\mathrm{eff}}P\right)\right\rangle = \frac{1}{M}\left\langle \mathrm{Tr}\left(PS_{\mathrm{eff}}^{\dagger}QS_{\mathrm{eff}}P\right)\right\rangle, \quad \text{(S38)}$$

where we introduced the projectors $P = \mathbb{1}_{\mathrm{M}}$ and $Q = \mathbb{1}_{\tilde{\mathrm{M}}} - P = \mathbb{1}_{\mathrm{M'}}$, and used the fact that the effective scattering matrix $S_{\mathrm{eff}}$ is unitary, unlike $S$. Combining this with the orthogonality condition $P \cdot Q = 0$ and the expression (S4), we find

$$A = \frac{1}{M}\left\langle \mathrm{Tr}\left[Pt^{\dagger}S_0^{\dagger}[1 - r'^{\dagger}S_0^{\dagger}]^{-1}t'^{\dagger}Qt'[1 - S_0 r']^{-1}S_0 tP\right]\right\rangle. \quad \text{(S39)}$$

This expression differs from Eq. (S36) only by the projectors. Selecting the rainbow diagrams, it simplifies to

$$A = \frac{\mathrm{Tr}(tPt^{\dagger})\mathrm{Tr}(t'^{\dagger}Qt')}{M_{\mathrm{in}}\tilde{M}}\sum_{n=0}^{\infty}\left[\frac{\mathrm{Tr}(r'^{\dagger}r')}{\tilde{M}}\right]^{n} = \frac{\Gamma\Gamma' M'}{\tilde{M} - \mathrm{Tr}(r'^{\dagger}r')} = \frac{a}{1 + a/\Gamma}. \quad \text{(S40)}$$

In Fig. S2, we compare the theoretical predictions (S37) and (S40) for the mean transmission and mean absorption with numerical simulations of transport through a chaotic absorbing cavity using random matrix modeling of the effective Hamiltonian. Excellent agreement is found over a wide range of the absorption-to-radiative loss ratio $a/\Gamma$.

### S2.E Equations for the edges of the eigenvalue spectrum

The bounds $z^*$ of the TMT eigenvalue distribution are determined by the condition $\partial_z g_T(z^*) = \infty$. From Eq. (S20), this condition is equivalent to $\partial_z \Sigma_{\mathrm{in}}^{+}|_{z^*} = \infty$, which in turn implies $\partial_z \Sigma_{\mathrm{tar}}^{+}|_{z^*} = \infty$, given the symmetry $\Sigma_{\mathrm{in}}^{+}(z, m_{\mathrm{in}}, m_{\mathrm{tar}}) = \Sigma_{\mathrm{tar}}^{+}(z, m_{\mathrm{tar}}, m_{\mathrm{in}})$. This symmetry ensures that exchanging $m_{\mathrm{in}}$ and $m_{\mathrm{tar}}$ does not alter the support of the non-zero TMT eigenvalue distribution. Differentiating Eq. (S27) and using the conditions $\partial_{\Sigma_{\mathrm{tar}}^{+}} z|_{z^*} = \partial_{\Sigma_{\mathrm{in}}^{+}} z|_{z^*} = 0$, we arrive at the equation

$$\frac{\partial_{\Sigma_{\mathrm{in}}^{+}} F_{\mathrm{in}}(z^*)}{\partial_{\Sigma_{\mathrm{in}}^{+}} F_{\mathrm{tar}}(z^*)} = \frac{\partial_{\Sigma_{\mathrm{tar}}^{+}} F_{\mathrm{in}}(z^*)}{\partial_{\Sigma_{\mathrm{tar}}^{+}} F_{\mathrm{tar}}(z^*)}, \quad \text{(S41)}$$

which can be explicitly expressed using Eqs. (S28) and (S29). The largest solution $z^*$ of the system of equations composed of $F_{\mathrm{in}}(z^*) = 0$, $F_{\mathrm{tar}}(z^*) = 0$, and Eq. (S41) defines the upper bound of the TMT distribution, while the smallest solution defines the lower bound.

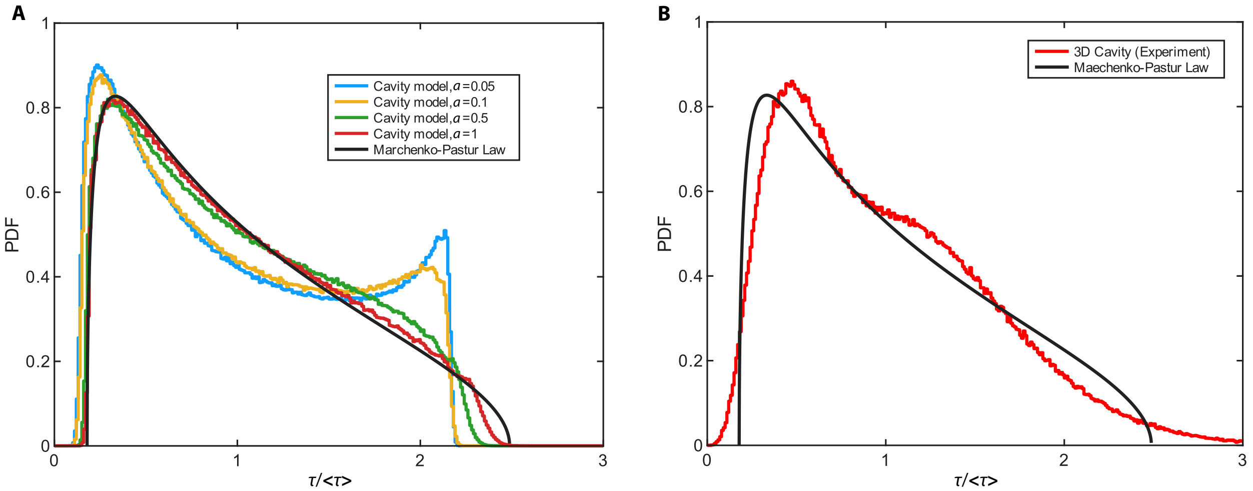


**Figure S3**: **Probability distributions of normalized TMT eigenvalues from cavity model simulations and 3D cavity experiments and comparison with Marchenko-Pastur Law.** The PDF of $\tau/\langle\tau\rangle$ using **(A)** random matrix theory modeling, for cavity wave simulations, for coupling parameter $\Gamma = 0.6$ and different absorption factors $a$, and **(B)** 3D cavity experiment (red line). The corresponding Marcenko-Pastur law is indicated in subfigures **(A,B)** with a black solid line. In all cases, the input/target ratio is $m_{\rm in} = 1/4$, $m_{\rm tar} = 3/4$. In the simulations, we have used GOE effective Hamiltonians $H_0$ of size $N = 600$ while the number of channels was $M = 160$.

## S3 Marcenko-Pastur Law for 3D reverberation chambers

When the mesoscopic correlations encoded in the matrix elements of the $M_{\text{tar}} \times M_{\text{in}}$ rectangular matrix $\tilde{S}$ are washed out by excess absorption, its elements can be treated as uncorrelated identically distributed random variables with zero mean. In this case the probability distribution $\mathcal{P}(x = \tau/\langle\tau\rangle)$ of the rescaled eigenvalues of the TMT matrix $T$ converges to the so-called Marcenko-Pastur (MP) law when $M_{\text{in}}, M_{\text{tar}} \to \infty$ while keeping $M_{\text{in}}/M_{\text{tar}}$ fixed. The MP law for non-zero eigenvalues takes the form

$$\mathcal{P}(x) = \frac{\sqrt{(x^+ - x)(x - x^-)}}{2\pi(m_{\text{in}}/m_{\text{tar}})x}, \tag{S42}$$

where $x^\pm = (1 \pm \sqrt{m_{\text{in}}/m_{\text{tar}}})^2$. This result holds for an arbitrary distribution with zero mean and equal variance of the elements of the rectangular matrix $\tilde{S}$. Hence, it also holds for the Gaussian random character expected for multiple scattered waves. The MP law has also been reproduced from our diagrammatic approach presented in Section II in the limiting case of lossy cavities ($a > 1$) or strong incomplete channel control ($m_{\text{in}}, m_{\text{tar}} \ll 1$).

Figure S3(**A**) shows the evolution of $\mathcal{P}(x)$ towards the MP distribution (solid black line) in the case of chaotic cavities simulated using a random matrix theory modeling (with coupling parameter $\Gamma = 0.6$), as losses are increased (the color lines indicate different $a$-values). Figure S3(**B**) reports the experimental results (red line) of $\mathcal{P}(x)$ for the reverberation cavity of Fig. 1(**C**) together with the MP law (solid black line). The cases depicted in Figs. S3(**A,B**) correspond to $m_{\text{in}} = 1/4$ and $m_{\text{tar}} = 3/4$.

Other representative $m_{\text{in}}, m_{\text{tar}}$ configurations are depicted in the subfigures Fig. S4(**A-C**). In these figures, we report the probability density function of TMT eigenvalues extracted from the measured $T$ matrices for the 3D cavity (blue lines) and the cavity simulations using a random matrix theory modeling (red lines). The corresponding diagrammatic results (which match the MP law) are also shown in these figures with black lines. In all cases, a nice agreement is observed.

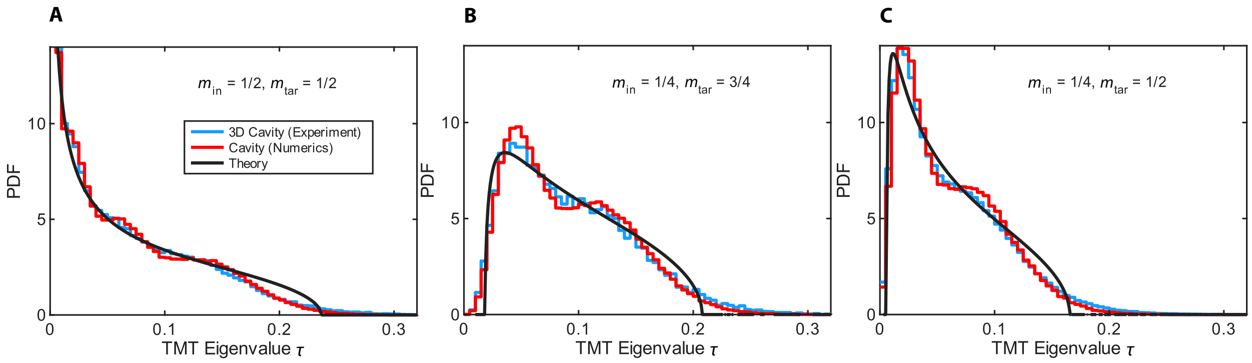


**Figure S4**: **TMT eigenvalue distributions in a 3D lossy chaotic cavity: experiment, simulation, and diagrammatic theory.** The probability density function (PDF) of the TMT eigenvalues of a 3D lossy chaotic cavity with $M = 8$ channels. The blue, red, and black lines in each subfigure indicate the experimental measurement of 3D cavity, the cavity simulations using a GOE modeling for the effective Hamiltonians (of dimension $N = 100$ and channel number $M = 8$), and the predictions of the diagrammatic approach, respectively. Different input and targeted combinations are shown: **(A)** $m_{\text{in}} = 1/2$ and $m_{\text{tar}} = 1/2$; **(B)** $m_{\text{in}} = 1/4$ and $m_{\text{tar}} = 3/4$; **(C)** $m_{\text{in}} = 1/4$ and $m_{\text{tar}} = 1/2$. In the simulation and theory, we used a coupling parameter $\Gamma \approx 0.95$ and an absorption factor $a \approx 6.3$, corresponding to a mean total absorption $A = a/(1 + a/\Gamma) \approx 0.8$.

## REFERENCES


1. L. Carneiro de Souza, C. H. de Souza Lopes, R. de Cassia Carlleti dos Santos, A. Cerqueira Sodré Jr., L. L. Mendes, A study on propagation models for 60 GHz signals in indoor environments. *Front. Commun. Netw.* **2**, 757842 (2022).

2. A. de Jesus Torres, L. Sanguinetti, E. Björnson, Electromagnetic interference in RIS-aided communications. *IEEE Wirel. Commun. Lett.* **11**, 668–672 (2022).

3. H. Cao, A. P. Mosk, S. Rotter, Shaping the propagation of light in complex media. *Nat. Phys.* **18**, 994–1007 (2022).

4. S. Gigan, O. Katz, H. B. de Aguiar, E. R. Andresen, A. Aubry, J. Bertolotti, E. Bossy, D. Bouchet, J. Brake, S. Brasselet, Y. Bromberg, H. Cao, T. Chaigne, Z. Cheng, W. Choi, T. Čižmár, M. Cui, V. R. Curtis, H. Defienne, M. Hofer, R. Horisaki, R. Horstmeyer, N. Ji, A. K. La Violette, J. Mertz, C. Moser, A. P. Mosk, N. C. Pégard, R. Piestun, S. Popoff, D. B. Phillips, D. Psaltis, B. Rahmani, H. Rigneault, S. Rotter, L. Tian, I. M. Vellekoop, L. Waller, L. Wang, T. Weber, S. Xiao, C. Xu, A. Yamilov, C. Yang, H. Yılmaz, Roadmap on wavefront shaping and deep imaging in complex media. *J. Phys. Photonics* **4**, 042501 (2022).

5. M. Di Renzo, A. Zappone, M. Debbah, M.-S. Alouini, C. Yuen, J. de Rosny, Smart radio environments empowered by reconfigurable intelligent surfaces: How it works, state of research, and the road ahead. *IEEE J. Sel. Areas Commun.* **38**, 2450–2525 (2020).

6. F. Xia, K. Kim, Y. Eliezer, S. Han, L. Shaughnessy, S. Gigan, H. Cao, Nonlinear optical encoding enabled by recurrent linear scattering. *Nat. Photonics* **18**, 1067–1075 (2024).

7. L. Chen, T. Kottos, S. M. Anlage, Perfect absorption in complex scattering systems with or without hidden symmetries. *Nat. Commun.* **11**, 5826 (2020).

8. W. R. Sweeney, C. W. Hsu, A. D. Stone, Theory of reflectionless scattering modes. *Phys. Rev. A* **102**, 063511 (2020).

9. X. Jiang, S. Yin, H. Li, J. Quan, H. Goh, M. Cotrufo, J. Kullig, J. Wiersig, A. Alù, Coherent control of chaotic optical microcavity with reflectionless scattering modes. *Nat. Phys.* **20**, 109–115 (2024).

10. C.-Z. Wang, J. Guillamon, W. Tuxbury, U. Kuhl, T. Kottos, Nonlinearity-induced scattering zero degeneracies for spectral management of coherent perfect absorption in complex systems. *Phys. Rev. Appl.* **22**, 064093 (2024).

11. A. Goïcoechea, J. Hüpfl, S. Rotter, F. Sarrazin, M. Davy, Detecting and focusing on a nonlinear target in a complex medium. *Phys. Rev. Lett*. **134**, 183802 (2025).

12. C. Guo, J. Li, M. Xiao, S. Fan, Singular topology of scattering matrices. *Phys. Rev. B* **108**, 155418 (2023).

13. J. Sol, A. Alhulaymi, A. D. Stone, P. Del Hougne, Reflectionless programmable signal routers. *Sci. Adv.* **9**, eadf0323 (2023).

14. U. Kuhl, M. Martínez-Mares, R. Méndez-Sánchez, H.-J. Stöckmann, Direct processes in chaotic microwave cavities in the presence of absorption. *Phys. Rev. Lett.* **94**, 144101 (2005).

15. V. Gopar, M. Martínez-Mares, R. Méndez-Sánchez, Chaotic scattering with direct processes: A generalization of Poisson's kernel for non-unitary scattering matrices. *J. Phys. A Math. Theor.* **41**, 015103 (2007).

16. G. Báez, M. Martínez-Mares, R. Méndez-Sánchez, Absorption strength in absorbing chaotic cavities. *Phys. Rev. E* **78**, 036208 (2008).

17. S. F. Liew, S. M. Popoff, A. P. Mosk, W. L. Vos, H. Cao, Transmission channels for light in absorbing random media: From diffusive to ballistic-like transport. *Phys. Rev. B* **89**, 224202 (2014).

18. P. W. Brouwer, Generalized circular ensemble of scattering matrices for a chaotic cavity with nonideal leads. *Phys. Rev. B* **51**, 16878–16884 (1995).

19. D. V. Savin, Y. V. Fyodorov, H.-J. Sommers, Reducing nonideal to ideal coupling in random matrix description of chaotic scattering: Application to the time-delay problem. *Phys. Rev. E* **63**, 035202 (2001).

20. Y. V. Fyodorov, S. Suwunnarat, T. Kottos, Distribution of zeros of the *S*-matrix of chaotic cavities with localized losses and coherent perfect absorption: Non-perturbative results. *J. Phys. A. Math. Theor* **50**, 30LT01 (2017).

21. A. Goetschy, A. Stone, Filtering random matrices: The effect of incomplete channel control in multiple scattering. *Phys. Rev. Lett.* **111**, 063901 (2013).

22. R. McIntosh, A. Goetschy, N. Bender, A. Yamilov, C. W. Hsu, H. Yılmaz, H. Cao, Delivering broadband light deep inside diffusive media. *Nat. Photonics* **18**, 744–750 (2024).

23. N. Bender, A. Yamilov, A. Goetschy, H. Yılmaz, C. W. Hsu, H. Cao, Depth-targeted energy delivery deep inside scattering media. *Nat. Phys.* **18**, 309–315 (2022).

24. L. Shaughnessy, R. E. McIntosh, A. Goetschy, C. W. Hsu, N. Bender, H. Yılmaz, A. Yamilov, H. Cao, Multiregion light control in diffusive media via wavefront shaping. *Phys. Rev. Lett.* **133**, 146901 (2024).

25. A. Z. Genack, Y. Huang, A. Maor, Z. Shi, Velocities of transmission eigenchannels and diffusion. *Nat. Commun.* **15**, 2606 (2024).

26. K. Joshi, I. Kurtz, A. Genack, Ohm's law lost and regained: Observation and impact of transmission and velocity zeros. *Nat. Commun.* **15**, 10616 (2024).

27. Z. Shi, A. Z. Genack, Transmission eigenvalues and the bare conductance in the crossover to Anderson localization. *Phys. Rev. Lett.* **108**, 043901 (2012).

28. T. Kottos, H. Schanz, Quantum graphs: A model for quantum chaos. *Physica E* **9**, 523–530 (2001).

29. T. Kottos, U. Smilansky, Quantum graphs: A simple model for chaotic scattering. *J. Phys. A. Math. Gen.* **36**, 3501 (2003).

30. Z. Pluhař, H. A. Weidenmüller, Universal chaotic scattering on quantum graphs. *Phys. Rev. Lett.* **110**, 034101 (2013).

31. Z. Pluhař, H. Weidenmüller, Universal quantum graphs. *Phys. Rev. Lett.* **112**, 144102 (2014).

32. H. Schanz, T. Kottos, Scars on quantum networks ignore the Lyapunov exponent. *Phys. Rev. Lett.* **90**, 234101 (2003).

33. C.-Z. Wang, U. Kuhl, A. Dowling, H. Schanz, T. Kottos, Bound states in the continuum induced via local symmetries in complex structures. *Phys. Rev. Appl.* **22**, 014010 (2024).

34. N. E. Hurt, Mathematical Physics of Quantum Wires and Devices: From Spectral Resonances to Anderson Localization (Springer Science & Business Media, 2000), vol. 506.

35. P. Kuchment, Graph models for waves in thin structures. *Waves Random Media* **12**, R1–R24 (2002).

36. P. Kuchment,Quantum graphs: I. Some basic structures. *Waves Random Media* **14**, S107–S128 (2003).

37. G. Berkolaiko, P. Kuchment, *Introduction to Quantum Graphs* (American Mathematical Society, 2013), vol. 186.

38. C. Brewer, S. C. Creagh, G. Tanner, Elastodynamics on graphs—Wave propagation on networks of plates. *J. Phys. A. Math. Theor.* **51**, 445101 (2018).

39. T. Lawrie, G. Tanner, C. Dimitrios, A quantum graph approach to metamaterial design. *Sci. Rep.* **12**, 18006 (2022).

40. J. J. M. Verbaarschot, H. A. Weidenmüller, M. R. Zirnbauer, Grassmann integration in stochastic quantum physics: The case of compound-nucleus scattering. *Phys. Rep.* **129**, 367–438 (1985).

41. Y. V. Fyodorov, H.-J. Sommers, Statistics of resonance poles, phase shifts and time delays in quantum chaotic scattering: Random matrix approach for systems with broken time-reversal invariance. *J. Math. Phys.* **38**, 1918–1981 (1997).

42. C. W. J. Beenakker, Random-matrix theory of quantum transport. *Rev. Mod. Phys.* **69**, 731–808 (1997).

43. S. M. Popoff, A. Goetschy, S. Liew, A. D. Stone, H. Cao, Coherent control of total transmission of light through disordered media. *Phys. Rev. Lett.* **112**, 133903 (2014).

44. C. W. Hsu, S. F. Liew, A. Goetschy, H. Cao, A. Douglas Stone, Correlation-enhanced control of wave focusing in disordered media. *Nat. Phys.* **13**, 497–502 (2017).

45. P. Boucher, A. Goetschy, G. Sorelli, M. Walschaers, N. Treps, Full characterization of the transmission properties of a multi-plane light converter. *Phys. Rev. Res.* **3**, 023226 (2021).

46. N. Bender, A. Goetschy, C. W. Hsu, H. Yilmaz, P. J. Palacios, A. Yamilov, H. Cao, Coherent enhancement of optical remission in diffusive media. *Proc. Natl. Acad. Sci. U.S.A.* **119**, e2207089119 (2022).

47. L. Erdős, T. Krüger, Y. Nemish, Scattering in quantum dots via noncommutative rational functions. *Ann. Henri Poincaré* **22**, 4205–4269 (2021).

48. V. A. Marchenko, L. A. Pastur, Distribution of eigenvalues for some sets of random matrices. *Math. USSR Sb.* **1**, 457–483 (1967).

49. G. Gradoni, M. Richter, S. Phang, S. B. Fedeli, U. Kuhl, O. Legrand, A. Ishimaru, "Statistical model for MIMO propagation channel in cavities and random media," in *2020 XXXIIIrd General Assembly and Scientific Symposium of the International Union of Radio Science* (IEEE, 2020), pp. 1–4.

50. P. W. Brouwer, C. W. J. Beenakker, Voltage-probe and imaginary-potential models for dephasing in a chaotic quantum dot. *Phys. Rev. B* **55**, 4695–4702 (1997).

51. Y. V. Fyodorov, D. V. Savin, H.-J. Sommers, Scattering, reflection and impedance of waves in chaotic and disordered systems with absorption. *J. Phys. A. Math. Gen.* **38**, 10731–10760 (2005).

52. P. W. Brouwer, C. W. J. Beenakker, Diagrammatic method of integration over the unitary group, with applications to quantum transport in mesoscopic systems. *J. Math. Phys.* **37**, 4904–4934 (1996).

53. P. A. Mello, Averages on the unitary group and applications to the problem of disordered conductors. *J. Phys. A. Math. Gen.* **23**, 4061–4080 (1990).
